# Wnt/GSK‐3β mediates posttranslational modifications of FLYWCH1 to regulate intestinal epithelial function and tumorigenesis in the colon

**DOI:** 10.1002/cac2.12625

**Published:** 2024-10-30

**Authors:** Sheema Almozyan, Roya Babaei‐Jadidi, Abrar Aljohani, Sepideh Youssefi, William Dalleywater, Prerna Kadam, Bradley Spencer‐Dene, Emad Rakha, Mohammad Ilyas, Abdolrahman Shams Nateri

**Affiliations:** ^1^ Cancer Genetics & Stem Cell Group BioDiscovery Institute School of Medicine University of Nottingham Nottingham Nottinghamshire UK; ^2^ Department of Cell Therapy and Immunobiology Research & Innovation Centre King Faisal Specialist Hospital & Research Centre RUH Riyadh Saudi Arabia; ^3^ Respiratory Medicine BioDiscovery Institute School of Medicine University of Nottingham Nottingham Nottinghamshire UK; ^4^ University of Nottingham and Nottingham University City Hospital School of Medicine Nottingham Nottinghamshire UK; ^5^ Department of Clinical Laboratory Sciences College of Applied Medical Sciences Taif University Taif Saudi Arabia; ^6^ Histopathology, Queens Medical Centre School of Medicine University of Nottingham Nottingham Nottinghamshire UK; ^7^ Experimental Histopathology The Francis Crick Institute London UK; ^8^ Non‐Clinical Histology Bioimaging GSK Stevenage Hertfordshire UK; ^9^ Academic Unit of Translational Medical Sciences School of Medicine University of Nottingham Nottingham Nottinghamshire UK

**List of abbreviations**
CHX: cycloheximide
Co‐IP: co‐immunoprecipitation
CRC: colorectal cancer
ELISA: enzyme‐linked immunosorbent assay
FLYWCH1: FLYWCH‐Type Zinc Finger 1
GSK‐3β: glycogen synthase kinase‐3β
IF: immunofluorescence
IHC: immunohistochemistry
ISC: intestinal stem cell
ISH: In‐situ hybridisation
PTM: post‐translational modification
RMSD: Root mean square deviation
TMA: tissue microarray
Wnt3A/Rspo: Wnt‐3A and R‐spondin1

The intestinal epithelium undergoes rapid renewal, with the entire epithelial layer replaced within five days. Intestinal stem cells (ISCs), located in the intestinal crypts, generate all differentiated cell types necessary for intestinal function. Key signalling pathways involved in stem cell maintenance include Wnt, Notch, Hedgehog, and BMP. Wnt signalling, primarily driven by crypt cells, creates a signalling gradient to maintain homeostasis [1]. However, nuclear β‐catenin, the key regulator of Wnt signalling, correlates positively with tumorigenesis. While crypt base cells also exhibit high levels of nuclear β‐catenin, the regulatory mechanism in normal tissue versus tumor remains unclear [1]. FLYWCH‐Type Zinc Finger 1 (FLYWCH1), an uncharacterised transcription factor, binds unphosphorylated‐β‐catenin [2], is associated with H3K9me3 in (peri)centromeric chromatin [3], and colocalizes with γ‐H2AX foci [4]. While its deletion is embryonically lethal in mice [5], the specific role and regulation of FLYWCH1 in tissue homeostasis and tumorigenesis remain unclear.

This study investigates the role of FLYWCH1 in intestinal stem cell regulation and its impact on colorectal cancer. We hypothesize that FLYWCH1 directly influences ISC function by modulating critical signalling pathways, thereby playing a significant role in the initiation and progression of colorectal cancer (CRC).

To assess the significance of FLYWCH1 expression in intestinal tissue homeostasis, we first examined its expression in murine tissues. Data from BioGPS (http://biogps.org) and the mouse gene expression database indicate varying tissue expression of *Flywch1*, with the highest level observed in the brain (Supplementary Figure S1). To confirm this, we conducted in‐situ hybridisation (ISH) analysis to identify distinct cell type‐specific expression patterns in the brain and intestinal tissues. ISH was performed using a Digoxigenin‐labelled antisense‐RNA probe for *Flywch1* mRNA on representative brain, liver and intestinal sections from 16‐week‐old wild‐type mice (Supplementary Figure S2A‐G). We observed high expression of *Flywch1* in cells located alongside the ISC marker Olfactomedin‐4 (*Olmf4*)‐positive cells, while *Flywch1* was not detectable in the differentiated epithelial cells of the intestinal villi (Supplementary Figure S2D‐E). In addition, we examined the differential expression of FLYWCH1 during carcinogenesis, initially in the intestine of *Apc* ^Min+/−^ mice, which harbour tumors and adjacent non‐tumor regions. *Flywch1* expression was substantially downregulated in intestinal neoplastic crypts compared to normal crypts (Supplementary Figure S2F‐G). This is consistent with FLYWCH1 expression in human CRC tissues (Figure 1A‐B, Supplementary Table S1). Collectively, these studies suggest a potential role for FLYWCH1 in ISC and the development of CRC.

**FIGURE 1:**
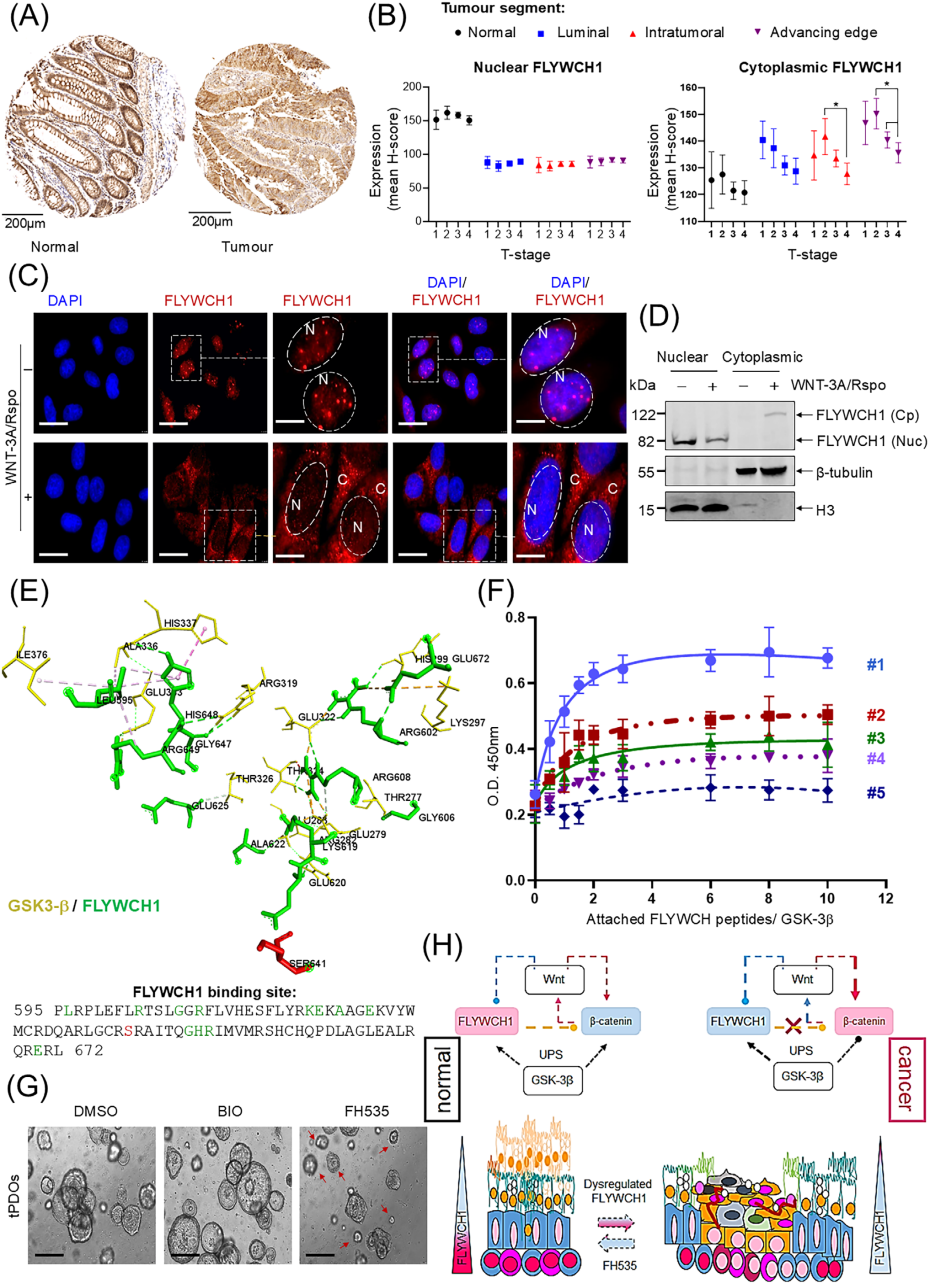
Crosstalk between FLYWCH1 and β‐Catenin through Wnt activation and GSK3‐β, and its role in maintaining intestinal stem cell homeostasis and tumorigenesis modulation. (A) Representative images of normal (left panel) and tumor (right panel) tissues from tissue microarrays (TMAs) of CRC patient samples showing differential expression of FLYWCH1 via immunohistochemistry (IHC). Scale bar: 200 µm. (B) Nuclear (left panel) and cytoplasmic (right panel) FLYWCH1 protein expression in epithelial cells across different tumor regions–; normal, luminal, intratumoral, and advancing edge– was quantified using the H‐score system. TMA cores with less than 15% intact epithelial tumor tissue were excluded from the analysis. p‐values are provided in Supplementary Table S1. (C) Immunofluorescence staining demonstrates the effect of Wnt‐3A/Rspo on the cellular distribution and expression pattern of FLYWCH1. Dotted lines indicate enlarged cells, with “N” representing the nucleus and “C” representing the cytoplasm. Scale bars for the original images: 7.5 µm. Scale bars for the zoomed‐in images: 50 µm. (D) Western blot (WB) analysis of nuclear and cytoplasmic FLYWCH1distribution following Wnt‐3A/Rspo treatment. (E) 3D interaction model showing GSK‐3β (yellow) and FLYWCH1 (green) binding, with the amino acid residue sequences of the FLYWCH1 C‐terminus binding site. The serine amino acid in the FLYECH1 C‐terminus docking site with GSK‐3β is highlighted in red. (F) ELISA results showing the binding of GSK‐3β antibody to wells containing wild‐type (#2) and mutant (#3) C‐terminal FLYWCH1‐GSK‐3β binding peptides, compared with wild‐type (#4) and mutant (#5) N‐terminal FLYWCH1 peptides and the control Axin‐GSK‐3β binding peptide (#1). Data points represent the average of triplicates from three independent experiments. (G) Representative images of day 5 tumor PDOs treated with DMSO, BIO (5 µmol/L), and FH535 (18 µmol/L) for two days. Organoid size is reduced with FH535 treatment compared to BIO treatment and DMSO‐treated patients‐derived tumor organoids (PDOs), as indicated by the differences marked with arrowheads. Scale bars: 7.5 µm. (H) Schematic model illustrating the mechanism by which activated Wnt and GSK‐3β promote the post‐translational modification of FLYWCH1. This leads to the suppressive activity of FLYWCH1 on nuclear β‐catenin and proliferation in cancer cells through cytoplasmic translocation and degradation of FLYWCH1 protein within cancer cells. The antiproliferative effect of FH535, which induces FLYWCH1 expression in tumor PDOs, suggests its potential therapeutic value in CRC treatment. Abbreviations: 3D, three‐dimensional; C, Cytoplasmic; DAPI, 4’,6‐diamidino‐2‐phenylindole; DMSO, Dimethyl sulfoxide; EIASA, Enzyme‐linked immunosorbent assay; FLYWCH1, FLYWCH‐Type Zinc Finger 1; GSK‐3β, Glycogen synthase kinase‐3 beta; H3, Histone H3; kDa, Kilodalton; N, Nuclear; PDO, Patient‐derived organoid; Rspo, R‐spondin; WB, Western blot.

To investigate the specific role of FLYWCH1 in ISCs and CRC, we performed loss‐of‐function experiments by expressing *Flywch1*‐gRNA in Cas9‐expressing intestinal organoids (Supplementary Figure S3A). This was followed by Southern blot analysis to evaluate the establishment of *Flywch1*‐knockout (*Flywch1* ^K/O^) organoid lines (Supplementary Figure S3B). The results indicated that *Flywch1* ^K/O^ organoids, displayed larger size and increased crypt budding compared to the normal control (Supplementary Figure S4A‐E). Additionally, the expression of cycling stem cell marker (*Lgr5*, *Olmf4*) and enteroendocrine cell mediators (*Ngn3*, *Pax4*) expressions was increased in *Flywch1* ^K/O^ organoids, while the expression of quiescent markers (*Tert*, *Lrig1*) was reduced. *Atoh1* (a Paneth‐cell mediators) remained unchanged (Supplementary Figure S4F‐H). In contrast, overexpression of FLYWCH1 (FLYWCH1*^OE^*) significantly repressed CRC patient‐derived tumor organoids (PDOs) growth (Supplementary Figure S4I‐K), and cancer stem‐like cell markers (*CD44*, *LGR5*) (Supplementary Figure S4L). These observations suggest that FLYWCH1 play a key role in intestinal homeostasis and tumorigenesis.

To investigate the relationship between FLYWCH1 expression and Wnt signalling, we evaluated the impact of FLYWCH1 on the expression of Wnt target genes in CRC cells. We used qRT‐PCR to analyse both *FLYWCH1*‐knockout (*FLYWCH1* ^K/O^) (Supplementary Figure S5A‐C) and FLYWCH1‐overexpressing (FLYWCH1^OE^) SW620 cells (Supplementary Figure S5D‐G). We assessed the expression of a subset of Wnt‐associated gene, including frizzled genes (FZD5, FZD6, FZD7), WNT1, WNT6, lipoprotein receptor‐related protein genes (LRP5, LRP6), homeobox protein CDX2, fibroblast growth factor 4 (FGF4), B‐cell CLL/lymphoma 9 (BCL9), and the cell surface adhesion receptor CD44 (Supplementary Figure S5H‐I). In line with our previous report [2], differential expression of FLYWCH1 influenced the expression of genes associated with Wnt pathway.

Additionally, we analysed FLYWCH1 expression in various CRC and normal epithelial cell lines (TIG119, CCD841‐CoN). Immunofluorescence (IF) staining revealed that the nuclear foci formed by FLYWCH1 were more pronounced in TIG119 and CCD841‐CoN cells compared to the diffuse nuclear expression observed in CRC cells (Figure 1C, Top panels, Supplementary Figure S6A). After 24 hours treatment with Wnt‐3A and R‐spondin1 (Wnt3A/Rspo), which activate Wnt receptors, we observed the accumulation of unexpected FLYWCH1 foci in the cytoplasm of all tested cell lines (Figure 1C, Bottom panels, Supplementary Figure S6B), along with a decrease in the total level of nuclear FLYWCH1 protein (Figure 1D, Supplementary Figure S6C). However, treatment with FH535, an inhibitor of β‐catenin and PPAR that acts at the level of transcription of target genes, notably increased FLYWCH1, inhibited glycogen synthase kinase‐3β (GSK‐3β) and β‐catenin levels (Supplementary Figure S6D‐E), while treatment with the GSK‐3β‐specific β‐catenin phosphorylation sites inhibitor (BIO) inhibited FLYWCH1 expression (Supplementary Figure S6F). These results suggest an antagonistic relationship between FLYWCH1 and GSK‐3β/β‐catenin signalling, where aberrant Wnt pathway activity may induce a negative feedback loop to downregulate FLYWCH1 activity.

Previously, we reported that FLYWCH1 interacts with β‐catenin via its N‐terminal domain of β‐catenin, where GSK‐3β phosphorylates β‐catenin [2]. Here, we further investigated the potential crosstalk between FLYWCH1 and GSK‐3β. Our bioinformatics databases and molecular docking simulations confirmed potential binding sites and interaction orientations between FLYWCH1 and GSK‐3β (Figure 1E, Supplementary Video S1, Supplementary Figure S7A, and Supplementary Tables S2‐S4). We performed a comparative binding enzyme‐linked immunosorbent assay (ELISA) (Figure 1F, Supplementary Figure S7B) and co‐immunoprecipitation (Co‐IP) assay, which were attenuated upon Wnt3A/Rspo treatment (Supplementary Figure S7C). Our analysis revealed various post‐translational modifications (PTMs), including ubiquitination and GSK‐3β consensus sequence phosphorylation sites within FLYWCH1 amino acid residues (Supplementary Figure S8A). A cycloheximide (CHX) chase assay determined the time course of FLYWCH1 protein stability (Supplementary Figure S8B) and demonstrated ubiquitination‐mediated degradation (Supplementary Figure S8C). FLYWCH1‐IP‐based assays and BIO treatment further showed that GSK‐3β maintained nuclear ubiquitinated FLYWCH1, while Wnt activation and GSK‐3β phosphorylation of FLYWCH1 led to its ubiquitination and subsequent degradation (Supplementary Figure S8D). These data suggest that the crosstalk between nuclear FLYWCH1 and Wnt/GSK‐3β increases FLYWCH1 phosphorylation, which could be crucial for proteasome‐mediated FLYWCH1 destabilisation.

To investigate the therapeutic significance of FLYWCH1 and GSK‐3β crosstalk in CRC, we screened PDOs for growth with six different Wnt small molecule inhibitors and activators, including the GSK‐3β phosphorylation blocker BIO, QS11, WAY‐316606, WIK14, FH535, and a GSK‐3β inhibitor drug. After 72 hours, the WST‐1 proliferation assay showed that BIO induced the most growth, while FH535 resulted in lowest growth compared to control organoids and other drugs (Figure 1G, Supplementary Figure S9A).

The clinical significance of FLYWCH1/GSK‐3β crosstalk was further examined using a CRC tissue microarray (TMA) via immunohistochemistry (IHC) and H‐score analysis (Supplementary Figure S9B, Supplementary Table S5). H‐score analysis showed that while the nuclear GSK‐3β expression was not significantly different between normal and tumor samples, nuclear GSK‐3β significantly increased with tumor stage (Supplementary Figure S9B), contrasting with FLYWCH1 (Figure 1B, left panel). However, cytoplasmic GSK‐3β staining intensity decreased with tumor stage (Supplementary Figure S9C), similar to FLYWCH1 (Figure 1B, right panel) in tumor samples. Furthermore, patients with low cytoplasmic FLYWCH1 and high cytoplasmic GSK‐3β had the shortest overall survival (OS) (Supplementary Figure S10A‐B and Supplementary Table S6), while no significant differences were observed for nuclear FLYWCH1 or nuclear GSK‐3β (Supplementary Figure S10C‐D and Supplementary Table S6).

In conclusion, we propose a model (Figure 1H) in which FLYWCH1 acts as a negative regulator of Wnt/β‐catenin signalling, maintaining ISC homeostasis. Aberrant Wnt activation mediates the cytoplasmic translocation of FLYWCH1 protein, and further dysregulation of FLYWCH1 by GSK‐3β disrupts this complex, leading to uncontrolled β‐catenin activity during CRC development and progression. Our data, along with previous findings [6], suggest that FH535 might inhibit tumor growth by GSK‐3β/β‐catenin inhibition, inducing FLYWCH1 expression. Altogether, this highlights FLYWCH1 as a potential therapeutic target in Wnt/β‐catenin‐driven tumorigenesis. Recent studies have identified associations between FLYWCH1 and the Wnt/β‐catenin pathway, particularly with chromatin in DNA damage response and radioresistance [3, 4, 7‐10]. A more detailed analysis of FLYWCH1's role in ISC regulation and CRC, including its therapeutic potential and differential subcellular localization, is discussed in the “Extra discussion text” section of the Supplementary Materials. Therefore, future investigations targeting FLYWCH1 for clinical anti‐cancer treatment strategies and monitoring treatment efficiency are warranted.

### AUTHOR CONTRIBUTIONS

*Conception and design*: Sheema Almozyan and Abdolrahman Shams Nateri. *Development of methodology*: Sheema Almozyan, Abrar Aljohani, Sepideh Youssefi, Prerna Kadam, Roya Babaei‐Jadidi, Bradley Spencer‐Dene, Emad Rakha, and Abdolrahman Shams Nateri. *Acquisition of data*: Sheema Almozyan, Abrar Aljohani, Sepideh Youssefi, Prerna Kadam, William Dalleywater, Bradley Spencer‐Dene, and Roya Babaei‐Jadidi. *Analysis and interpretation of data*: Sheema Almozyan, Sepideh Youssefi, Prerna Kadam, William Dalleywater, Roya Babaei‐Jadidi, and Abdolrahman Shams Nateri. *Writing and revision of the manuscript*: Sheema Almozyan, William Dalleywater, and Abdolrahman Shams Nateri. *Administrative, technical, or material support*: Sheema Almozyan, Roya Babaei‐Jadidi, Mohammad Ilyas, Emad Rakha, and Abdolrahman Shams Nateri. *Supervisions*: Mohammad Ilyas, Emad Rakha, and Abdolrahman Shams Nateri. All authors have read, reviewed and agreed to the published version of the manuscript.

## CONFLICT OF INTEREST STATEMENT

The authors declare no potential conflicts of interest.

## FUNDING INFORMATION

This research was supported by the Medical Research Council (MRC) (grant number G0700763) and by the University of Nottingham, Nottingham, UK.

## ETHICS APPROVAL AND CONSENT TO PARTICIPATE

For patient‐derived organoids, CRC specimens were collected from the Nottingham Health Sciences Biobank (NHSB) at Queens Medical Centre, University of Nottingham (NHSB approval number: ACP000098 A Nateri CRC). Ethical approval and research and development approval for generating the TMA were obtained from the Local Research Ethics Committee and the Trust Research and Development office in Nottingham (Ethical approval reference number: 05/Q1605/66), respectively. Mice for organoid isolations were kept in a specific pathogen‐free condition (PPL number: P375A76FE). All experiments were conducted at the University of Nottingham in accordance with institutional biomedical service unit guidelines.

## Supporting information



Supporting Information

Supporting Information

## Data Availability

Data generated in the present study are available from the corresponding author upon request. The clinical information and gene expression data for FLYWCH/GSK‐3β studied in colorectal cancer TMA slides have been uploaded to https://doi.org/10.7910/DVN/KTAFUC.

## References

[1] Colozza G , Park SY , Koo BK . Clone wars: From molecules to cell competition in intestinal stem cell homeostasis and disease. Exp Mol Med. 2022;54(9):1367‐78. 36117218 10.1038/s12276-022-00854-5 PMC9534868

[2] Muhammad BA , Almozyan S , Babaei‐Jadidi R , Onyido EK , Saadeddin A , Kashfi SH , et al. FLYWCH1, a Novel Suppressor of Nuclear beta‐Catenin, Regulates Migration and Morphology in Colorectal Cancer. Mol Cancer Res. 2018;16(12):1977‐90. 30097457 10.1158/1541-7786.MCR-18-0262 PMC6277001

[3] Santos‐Barriopedro I , van Mierlo G , Vermeulen M . Off‐the‐shelf proximity biotinylation for interaction proteomics. Nat Commun. 2021;12(1):5015. 34408139 10.1038/s41467-021-25338-4 PMC8373943

[4] Almozyan S , Coulton J , Babaei‐Jadidi R , Nateri AS . FLYWCH1, a Multi‐Functional Zinc Finger Protein Contributes to the DNA Repair Pathway. Cells. 2021;10(4):889. 33924684 10.3390/cells10040889 PMC8069811

[5] Dickinson ME , Flenniken AM , Ji X , Teboul L , Wong MD , White JK , et al. High‐throughput discovery of novel developmental phenotypes. Nature. 2016;537(7621):508‐14. 27626380 10.1038/nature19356 PMC5295821

[6] Tu X , Hong D , Jiang Y , Lou Z , Wang K , Jiang Y , et al. FH535 inhibits proliferation and migration of colorectal cancer cells by regulating CyclinA2 and Claudin1 gene expression. Gene. 2019;690:48‐56. 30552982 10.1016/j.gene.2018.12.008

[7] Jun S , Jung YS , Suh HN , Wang W , Kim MJ , Oh YS , et al. LIG4 mediates Wnt signalling‐induced radioresistance. Nat Commun. 2016;7:10994. 27009971 10.1038/ncomms10994 PMC4820809

[8] Gupta R , Somyajit K , Narita T , Maskey E , Stanlie A , Kremer M , et al. DNA Repair Network Analysis Reveals Shieldin as a Key Regulator of NHEJ and PARP Inhibitor Sensitivity. Cell. 2018;173(4):972‐88 e23. 29656893 10.1016/j.cell.2018.03.050 PMC8108093

[9] Rispal J , Escaffit F , Trouche D . Chromatin Dynamics in Intestinal Epithelial Homeostasis: A Paradigm of Cell Fate Determination versus Cell Plasticity. Stem Cell Rev Rep. 2020;16(6):1062‐80. 33051755 10.1007/s12015-020-10055-0 PMC7667136

[10] Sheng X , Lin Z , Lv C , Shao C , Bi X , Deng M , et al. Cycling Stem Cells Are Radioresistant and Regenerate the Intestine. Cell Rep. 2020;32(4):107952. 32726617 10.1016/j.celrep.2020.107952 PMC7789978

