# Supplementary material for: Wnt/GSK‐3β mediates posttranslational modifications of FLYWCH1 to regulate intestinal epithelial function and tumorigenesis in the colon
Source: Cancer Commun (Lond). 2024 Oct 30;45(1):9–14. doi: 10.1002/cac2.12625 (PMC11758259; doi:10.1002/cac2.12625)
Supplement: Supplementary file 1 — Supporting Information [file CAC2-45-9-s001.docx]

**Supplementary Materials**

**Wnt/GSK-3β mediates posttranslational modifications of FLYWCH1 to regulate intestinal epithelial function and tumorigenesis in the** **colon**

Sheema Almozyan ^1, 2^, Roya Babaei-Jadidi ^1, 3^, Abrar Aljohani ^4, 5^, Sepideh Youssefi ^1^, William Dalleywater ^6^, Prerna Kadam ^1^, Bradley Spencer-Dene ^7, 8^, Emad Rakha ^4^, Mohammad Ilyas ^6^, Abdolrahman Shams Nateri ^1, 9 *^

^1^ Cancer Genetics & Stem Cell Group, BioDiscovery Institute, School of Medicine, University of Nottingham, Nottingham, Nottinghamshire, NG7 2RD, United Kingdom.

^2^ Department of Cell Therapy and Immunobiology, Research & Innovation Centre, King Faisal Specialist Hospital & Research Centre, Riyadh, RUH, 11211, Saudi Arabia.

^3^ Respiratory Medicine, BioDiscovery Institute, School of Medicine, University of Nottingham, Nottingham, Nottinghamshire, NG7 2RD, United Kingdom.

^4^ University of Nottingham and Nottingham University City Hospital, School of Medicine, Nottingham, Nottinghamshire, NG2 1AP, United Kingdom.

^5^ Department of Clinical Laboratory Sciences, College of Applied Medical Sciences, Taif University, Taif, 21944, Saudi Arabia.

^6^ Histopathology, Queens Medical Centre, School of Medicine, University of Nottingham, Nottingham, Nottinghamshire, NG7 2UH, United Kingdom.

^7^ Experimental Histopathology, The Francis Crick Institute, London, NW1 1AT, United Kingdom.

^8^ Non-Clinical Histology, Bioimaging, GSK, Stevenage, Hertfordshire, SG1 2NY, United Kingdom.

^9^ Academic Unit of Translational Medical Sciences, School of Medicine, University of Nottingham, Nottingham, Nottinghamshire, NG7 2UH, United Kingdom.

^*^ Corresponding author:

Abdolrahman Shams Nateri, BioDiscovery Institute, School of Medicine, University of Nottingham, Nottingham, Nottinghamshire, NG7 2RD, United Kingdom, Email: [a.nateri@nottingham.ac.uk](mailto:a.nateri@nottingham.ac.uk)

**Extra discussion text:**

**Specificity of FLYWCH1’s role in ISC, CRC and its potential as a therapeutic target.**

Our study highlights the unique role of FLYWCH1 as a transcriptional regulator, emerging as a crucial player in ISC function and CRC progression. We identified tissue-specific expression patterns of FLYWCH1, showing significant upregulation in ISC and downregulation in CRC tissues compared to normal colonic epithelium, a pattern distinct from other ISC regulators. Knockout of FLYWCH1 resulted in an increase in expression of cycling ISC and enteroendocrine progenitors/precursors mediators, suggesting its specificity compared to general ISC regulators. However, our data indicate that FLYWCH1 likely interacts with key components of the Wnt pathway and ISC regulators, such as GSK-3β and β-catenin. FLYWCH1 modulates Wnt signalling by regulating the interaction between β-catenin and TCF4, suggesting a novel regulatory role rather than a redundant one. We demonstrated how FLYWCH1 is post-translationally modified and regulated by Wnt signalling, supported by biochemical and molecular biology data. This suggests that FLYWCH1 forms a transcriptional regulatory mechanism responsive to Wnt signalling, defining stem cell identity in a Wnt-dependent manner. We plan to utilize CRC mouse models with conditional knockout of FLYWCH1 in ISC and/or enteroendocrine progenitors to assess its impact on intestinal homeostasis, tumor initiation, growth, and metastasis. Mechanistic studies will explore whether FLYWCH1 directly occupies its target genes or collaborates with other ISC regulators, such as ASCL2, in a regulatory circuitry, or interacts with signalling pathways like Notch and BMP. Techniques including single-cell RNA sequencing, chromatin immunoprecipitation sequencing (ChIP-seq), and proteomics will be employed to uncover FLYWCH1’s genomic binding sites, its role in transcriptional regulation, and to map its binding partners.

Building on our findings, we aim to validate FLYWCH1 as a therapeutic target in CRC through *in vitro* and *in vivo* studies. To explore the therapeutic potential of FLYWCH1/FH535, we will investigate the efficacy of FH535 *in vivo* using patient-derived xenografts (PDX) and CRC cell-derived xenograft models with stable FLYWCH1 overexpression or knockdown. Additionally, we will screen small molecule inhibitor libraries or siRNA-based therapies targeting FLYWCH1 in CRC cell lines and advanced patient-derived models to evaluate their efficacy in reducing tumor cell viability and stemness.

**Differences in FLYWCH1 expression between the nucleus and cytoplasm.**

The observed molecular weight difference of FLYWCH1 between the nucleus and cytoplasm can be attributed to several post-translational modifications (PTMs) differentially regulated in these compartments. Our data suggest that FLYWCH1 undergoes GSK-3β kinase-mediated phosphorylation and ubiquitination, which could contribute to the observed molecular weight variations. GSK-3β phosphorylation adds phosphate groups to the FLYWCH1 protein, increasing its apparent molecular weight, similar to its role in β-catenin phosphorylation. In contrast, the nuclear version of FLYWCH1 may be less phosphorylated, resulting in a lower molecular weight. Differential ubiquitination in the cytoplasm, possibly through mono- or polyubiquitination, may also contribute to the molecular weight difference compared to the nuclear counterpart. However, due to the lack of specific antibodies for phosphorylated and ubiquitinated FLYWCH1, we are unable to directly demonstrate the observed difference in the molecular weight of FLYWCH1 between the nucleus and the cytoplasm. Furthermore, FLYWCH1 might form complexes with other proteins or nucleic acids in the nucleus, affecting its migration during electrophoresis and altering the perceived molecular weight. These interactions may be less prominent or stable in the cytoplasm, contributing to the observed profile.

Nerveless, we studied the relationship between FLYWCH1 phosphorylation and ubiquitination by conducting co-immunoprecipitation assays followed by immunoblotting to examine whether phosphorylated FLYWCH1 associates with ubiquitination machinery components or undergoes ubiquitination (Supplementary Figure S7). Our in-silico computational algorithms and databases also suggest potential phospho- and ubiquitination sites in FLYWCH1 (Figure 1H and Supplementary Figure S6).

The translocation of key proteins like FLYWCH1 to the cytoplasm is often triggered by specific regulatory signals within the cell, including post-translational modifications such as phosphorylation or ubiquitination, which alters FLYWCH1’s localisation signals. We demonstrated translocation of FLYWCH1 is part of its dynamic regulation in response to cellular stimuli, such as Wnt signalling induced by WNT-3A and R-Spondin. This process enables cells to quickly adapt to changing conditions by adjusting the localisation and activity of FLYWCH1 in ISCs during differentiation or in CRC development. Such dynamic regulation ensures that cellular processes remain finely tuned and responsive.

Unfortunately, due to the lack of specific antibodies for phosphorylated and ubiquitinated forms of FLYWCH1, we were unable to identify the specific amino acid residues undergoing these modifications. Typically, this requires in-depth *in vitro* and *in vivo* analyses, beyond the scope of this study. In future studies, we plan to employ Mass Spectrometry (MS) for phospho-peptide mapping and use phosphoproteomics techniques such as LC-MS/MS to identify phosphorylation sites comprehensively. Additionally, we will introduce mutations at specific amino acid residues suspected of being phosphorylated to confirm their roles. Furthermore, we intend to investigate FLYWCH1 phosphorylation by screening protein-protein interactions, identifying interaction partners, and assessing changes in phosphorylation patterns upon knocking down or knocking out specific kinases or phosphatases.

**Supplementary Methods and Materials**

***In situ* hybridisation (ISH) assay**

Both sense and anti-sense FLYWCH1 RNA-probes were generated by cloning a short nucleotide sequence from human FLYWCH1 cDNA into a pcDNA3 vector. Both probes were conjugated with Digoxigenin using a DIG-RNA-labelling mix (Roche) and ISH-assay was carried out as described previously [1]. For ISH, photomicrographs of eight wild-type and ApcMin specimens from three mice were analysed. The percentages of positive crypts were calculated for 50 crypts each from normal, tumor and for adjacent non-tumor sections (total ~400 crypts). Crypts were scored as follows: "0" for no labelling, "1" for weak labelling, "2" for moderate labelling, and "3" for strong labelling. The H-score was calculated using the following formula: H-score = [(0 x % negative crypts) + (1 x % weak positive crypts) + (2 x % moderate positive crypts) + (3 x % strong positive crypts)]. Due to the consistently low variation among normal sections, the results for tumor and adjacent non-tumor crypt sections were normalized to the normal sections. Data are presented as the mean and standard deviation (StD).

**Mouse crypt isolation and organoid culture**

Intestinal crypts were isolated from C57BL/6J mice (4-6 weeks old) following previously described methods [2]. The samples were repeatedly washed with ice-cold PBS, opened longitudinally, and divided into 1 mm sections. Crypts were released from the epithelium by incubating the samples in 3 mmol/L ethylenediaminetetraacetic acid (EDTA) in phosphate-buffered saline (PBS) for 35 minutes at 4 °C. After counting and isolating the crypts by centrifugation, they were resuspended in Matrigel (BD Biosciences, Oxford, UK), seeded into a 48-well plate, and fed with fresh organoid medium comprising basal Advanced DMEM/F-12 medium, 2 mmol/L L-glutamine, 100 U/ml Penicillin/Streptomycin, and 10 mmol/L HEPES, supplemented with N2 (Fisher Scientific, 17502048) supplement (1X), B27 (Fisher Scientific, 12585-010) supplement (1X), 1 mmol/L N-acetylcysteine to stimulate cell proliferation, 50 ng/mL murine recombinant epidermal growth factor (mEGF, Fisher Scientific, PMG8043) to activate the EGF signaling pathway, 1 μg/mL R-Spondin 1 (in-house) as a Wnt agonist, and 100 ng/mL Noggin (in-house) to inhibit the BMP pathway. "Complete" DMEM is defined as Advanced DMEM/F-12 medium, 2 mmol/L L-glutamine, 100 U/ml Penicillin/Streptomycin, and 10 mmol/L HEPES. Organoids were maintained in a 37°C, 5% CO2 incubator, checked visually by an inverted microscope, and the organoid medium was replaced with fresh medium every 48 hours.

**Generation of organoid lines expressing FLYWCH1-gRNAs**

Initially, we generated organoid lines by transducing lentiviruses as outlined above, expressing human-optimized S. pyogenes Cas9 and GFP using the pLV hUbC-Cas9-T2A-GFP vector (Addgene: #53190). GFP and Cas9 expression were verified using fluorescent microscopy and western blotting assays, respectively. We then produced lentiviruses as outlined above, using the LV04 system (pLV-U6gRNA: hPGK-puro-2A-tBFP) Sanger-designed *FLYWCH1*-gRNA (Sigma; Sanger QP KO clones) (Supplementary Table S7), to target FLYWCH1 and LV40 negative control containing non-target gRNA (Sigma; CRISPR20V-1EA). Following puromycin treatments, genomic DNA from selected negative control and Flywch1-gRNA positive control lines was isolated by digestion in lysis buffer (100 mmol/L Tris-HCl, pH 8.5; 5 mmol/L EDTA; 0.1% sodium dodecyl sulfate (SDS); 100 mmol/L NaCl; and 50 µg/ml Proteinase K (Roche)) at 55°C. DNA was extracted from the lysates using phenol-chloroform and chloroform treatment, precipitated by isopropanol, and washed with 70% ethanol. The DNA pellet was dissolved in TE buffer (10 mmol/L Tris, pH 7.9; and 0.2 mmol/L EDTA). Approximately 10 µg of genomic DNA was digested with the single-cutter restriction enzymes SnaBI (Biolabs; R0130L) at position 280 and AhdI (Biolabs; R0584S) at position 6487 (Supplementary Figure S3). The DNA was fractionated on 0.8% agarose gels and transferred to GeneScreen nylon membranes (NEN DuPont). Membranes were hybridized with 32P-labeled DNA probes specific to the 5′- regions flanking the Puromycin. DNA labelling was performed using the random prime DNA labelling kit (Roche) and [32P] dCTP (PerkinElmer). Membranes were washed with 0.5x SSPE (1X SSPE is 0.18 mol/L NaCl, 10 mmol/L NaH2PO4, and 1 mmol/L EDTA, pH 7.7) and 0.5% SDS at 65°C and exposed to Kodak film at −80°C. The expected bands, approximately 6200 bp, were detected (Supplementary Figure S3). Due to limited resources, we could not sequence the targeted sequences versus negative controls. To avoid any biological and cellular changes that might be due to the exogenous Cas9-expression, we applied the parental organoids that are expressing Cas9-GFP (without expressing the gRNA) in our study. Furthermore, we used the commercial LV-gRNA non-target for sFlywch1-gRNA controls in our experiments, however, we have not observed any morphological or growth impact of these control lines with either wild-type parental or organoids expressing exogenous Cas9-GFP. In addition, CRISPR-Cas9 organoids experiments were performed in three independent lines and independent time where were possible.

**Tumor human organoids cultures**

The tumor and adjacent normal tissue samples of human colon were collected from the Nottingham Health Sciences Biobank (NHSB), Queens Medical Centre, and the University of Nottingham. All procedures were conducted following the Declaration of Helsinki and received approval from the local ethics committee. Organoids were cultured as previously established and characterized [3, 4]. Colonic crypts were re-suspended in Matrigel, seeded in a 48-well plate, and cultured in advanced DMEM/F-12 medium (Invitrogen, 12634-028) containing 2 mmol/L L-glutamine, 100 U/ml Penicillin/Streptomycin, and 10 mmol/L HEPES (Invitrogen, 15630-122). The medium was supplemented with 5 ml of Wnt-3a (conditioned medium), 2 ml of mNoggin (conditioned medium), 2 ml of R-Spondin1 as a Wnt agonist, B27 supplement (1 ml, 50X) (Invitrogen, 12587-010), N-acetylcysteine (125 μl, 1.25 mmol/L) (Sigma Aldrich, A9165), murine recombinant epidermal growth factor (mEGF) (25 μl, 100 μg/ml) (Invitrogen, PMG8043), TGF-beta inhibitor (A83-01) (5 μl, 10,000x, 5mmol/L), p38 inhibitor (SB202190) (5 μl, 30mmol/L), Primocin (100 μl, 500x), and Nicotinamide (250 μl, 10 mmol/L). Organoids were maintained in a 37°C, 5% CO2 incubator, checked visually by an inverted microscope, and the organoid medium replaced with fresh medium every 48 hours.

**Lentiviral production and organoids transduction**

For the production of lentivirus particles, HEK293-T cells were utilized. Confluent cells were co-transfected with 2 packaging plasmids (6 μg of pCMV delta R8.74 and 3 μg of pMDG2), along with 9 μg of the plasmid of interest, FLYWCH1-sgRNA (pLV-U6gRNA: hPGK-puro-2A-tBFP), the Cas9 and GFP expressing vector (pLV hUbC-Cas9-T2A-GFP), and FLYWCH1 and GFP expressing vector (pLVX-GFP- FLYWCH1-PuroR). Virus harvesting was conducted for 3 consecutive days, and virus particles were concentrated by ultracentrifugation for 4 hours at 22,000 rpm. Primary patient-derived and mouse intestinal organoids were infected through lentiviral transduction as previously described. In brief, lentiviral infection occurred after several washes with PBS. Organoids were gently dissociated by pipetting, the cells were then spun, and the pellets were directly infected with lentiviral particles diluted in the suitable organoid medium. The mixture was incubated for 6 hours at 37 °C. Subsequently, the pellet was embedded in Matrigel, and fresh organoid media were added.

**TMA Immunohistochemical analysis**

Immunohistochemistry for FLYWCH1 (1:500; Atlas Antibodies, HPA040753) and GSK-3B (1:400; Cell Signalling, 12456) was conducted in collaboration with the Histopathology Unit at City Hospital, University of Nottingham, using a CRC TMA containing 1000 primary colorectal cancers [5]. The TMA was generated from resection specimens’ of 1000 patients with CRC at City Hospital, University of Nottingham, who underwent surgery between 2008 and 2012. The average age of the patients was 68 years, and the mean follow-up time was 53 months. After completing the immunohistochemistry, the TMA slides were scanned at 20x magnification using the VENTANA DP 200 slide scanner (Roche, UK). The staining intensity was scored using the H-scoring system, calculated as previously described. TMA-stained sections were first checked by W.D. and E.R. to ensure the areas were defined correctly before scoring. Furthermore, cores with less than 15% invasive tumor tissue or intact (epithelial) tissue were excluded (i.e., not scored). The H-score ranged from 0 to 300, using the formula: (1 × % weakly stained nuclei) + (2 × % moderately stained nuclei) + (3 × % strongly stained nuclei). All cases were scored independently by S.A. and A.A., who were blinded to the patients’ pathological and outcome data. W.D. completed the data analysis and correlation with tumor segments and stages as provided in Supplementary Tables S1 and S5. These H-scores and key clinical data are included in the raw dataset (https://doi.org/10.7910/DVN/KTAFUC) and summarized in Supplementary Tables S1 and S5. To conduct survival analysis, the cytoplasmic and nuclear expression levels of each protein in tumor samples were normalised against the corresponding normal tissue controls for each patient. Expression levels in each compartment were categorized as "high" or "low" based on the mean expression: "low" was defined as expression below the mean, and "high" as expression equal to or above the mean. Summary statistics for each group are provided in the Supplementary Table S6. The association between expression levels (high vs. low) and overall survival was assessed for each marker. Statistical analysis, including survival analysis, was performed using IBM SPSS Statistics 23.

**Organoids immunohistochemistry**

Organoids were fixed in 4% paraformaldehyde (PFA) overnight at 4 °C. Then washed and resuspended in 2 % low-melting agarose prior to paraffin embedding. Later, 4 µm sections were cut and processed for immunohistochemistry analysis or Haematoxylin and Eosin (H&E) staining as previously reported [6].

**Immunofluorescence**

Samples were fixed in 4% PFA for 30 minutes at room temperature (RT), permeabilized using 0.1% BSA, 0.1% Triton X-100, 0.05% Tween-20 in PBS, blocked with 3% BSA (Bovine Serum Albumin prepared in PBS) for 1 hour at RT and stained overnight with appropriate concentration of primary antibody diluted in 2% BSA-PBS at 4 °C. This was followed by incubating with fluorescently conjugated secondary antibodies and imaged on a Zeiss LSM florescence microscope. Samples were counterstained with DAPI.

**Cell lines**

Four different immortalized human epithelial and colorectal cancer cell lines-CCD841-CoN, HCT116, SW480, SW620, and DLD-1-were utilized in this study, along with human embryonic kidney HEK293T cells. All cell lines were originally obtained from the American Type Culture Collection (ATCC). The HEK293T-HA-Rspo1-Fc cells, which stably express murine R-spondin1, were generously provided by Prof. Hans Clevers' laboratory. Additionally, human skin fibroblast cells TIG119 were a gift from Dr Axel Behrens' laboratory at the Institute of Cancer Research (ICR) in London [7]. Regular testing was conducted on the cell lines to ensure the absence of Mycoplasma contamination.

**Generation of *FLYWCH1*-knockout (*FLYWCH1*^K/O^), and overexpressing (FLYWCH1^O/E^) SW620 cells**

*FLYWCH1* sgRNA oligos targeting *Exon*1 of *FLYWCH1* gene was annealed and cloned into the pSpCas9(BB)-2A-Puro (PX459) vector from Addgene, following digestion with the BbsI restriction enzyme. After ligation and nucleotide sequencing analysis, SW620 cells were transfected with PX459 expressing the *FLYWCH1* gRNA (Supplementary Table S7). Puromycin-resistant, single-cell-derived colonies were then isolated and screened for FLYWCH1 expression. Overexpression of FLYWCH1 was carried out by transducing SW620 cells with the pLVX-GFP-FLYWCH1-PuroR lentivirus, following the same protocol as used for organoid generation.

**Sub-cellular fractionation**

Cells were treated with Wnt3a-CM, 5 μmol/L BIO or mock treated at the indicated time. Nuclear/cytosol protein separation was performed using Bio-Vision Nuclear/cytosol fractionation kit (K266-100, BioVision Inc, CA, USA). This kit consists of three main buffers: Cytosol Extraction Buffer A (CEB-A), Cytosol Extraction Buffer B (CEB-B), and Nuclear Extraction Buffer (NEB). First, CEB-A and NEB buffer mixtures were prepared separately by adding a protease inhibitor cocktail and DTT. For each ml of buffer, 2 μl of 1x protease inhibitor and 1 μl of DTT (1mol/L) were added and mixed gently. Following the manufacturer's protocol, cells were washed to remove any residual medium and collected by centrifugation. The pellet was first resuspended in 0.2 ml of the CEB-A buffer mix, vortexed for 20 seconds, and kept on ice for 10 minutes. After incubation, 11 μl of ice-cold CEB-B buffer was added to the mixture, kept on ice, and vortexed vigorously for 15 seconds. To separate the cytosolic protein, the sample was centrifuged at 16,000 g at 4°C for 5 minutes, and the supernatant (containing the cytosolic fraction) was transferred into a sterile, labelled Eppendorf tube. For the nuclear fraction, the pellet was resuspended in 100 μl of NEB buffer on ice and mixed vigorously by vertexing the sample every 5-10 minutes for 40 minutes. The tube was then centrifuged for 15 minutes at 16,000 g at 4°C, and the nuclear protein was collected into a sterile Eppendorf tube and stored at -20°C for short-term storage or -80°C for long-term storage. Nuclear and cytosolic proteins were then used in immunoblot analyses.

**Co-immunoprecipitation, ubiquitination (Ub) and western blotting assays**

IP, Ub, and WB assays were carried out as previously described [1, 8]. Using the following antibodies: FLYWCH1 (HPA041001; Prestige Antibodies), anti-GSK-3β (27C10; Cell-Signaling), anti-β-catenin (610154; BD), anti-GFP (3E6; Invitrogen), anti-Phospho - (Ser/Thr) antibody (AB117253; Abcam), p-c-MycT58/S62 (Cell-Signaling), anti-E-cadherin (610181; BD) and anti-β-actin (ab6276; Abcam).

**Competitive ELISA assay**

Wild-type and mutant variants of the C-terminal and N-terminal regions of FLYWCH1 peptides, along with a positive control peptide corresponding to the human Axin-binding domain to GSK-3β, were selected from the NCBI databases. The competitive ELISA assay was performed following the protocol described by Braitbard, at al. [9].

**Small-molecule modulators of the Wnt/β-catenin signaling pathway**

QS11 (3324; Tocris): an ARFGAP1 inhibitor, which regulates Wnt signaling by controlling LRP6 phosphorylation. WAY-316606 (4767; Tocris): an inhibitor of the secreted protein sFRP-1, which is the endogenous antagonist of the secreted glycoprotein Wnt. WIK14 (4855; Tocris): an inhibitor of the pathway via stabilization of Axin. FH535 (4344; Tocris): an inhibitor of β-catenin and PPAR, acting at the level of transcription of target genes, along with GSK-3B inhibitor (BIO). All compounds are dissolved in DMSO, respectively.

**WST-1 cell proliferation assay**

Following the manufacturer’s protocol, tumor organoid viability and proliferation in response to Wnt small molecule compounds was measured by WST-1 cell proliferation assay (Roche, UK). The assay is based on the cleavage of a tetrazolium salt, MTS, by mitochondrial dehydrogenase. Herein, control tumor organoids were treated with DMSO. Later, WST-1 reagent (11644807001, Roche) was added and incubated for 2 hours, the medium was then aspirated and transferred into another 96-well plate, and the absorbance was measured at a wavelength of 450 nm by Magellan software provided within TECAN (ELISA) reader. Tumor organoid medium was used as a blank control.

**RNA isolation, and RT-qPCR assay**

Organoids were dissociated from Matrigel by incubating with Cultrex Organoid Harvesting Solution (3700-100-01, R&D systems Bio-techne) for 1 hour at 4°C. Total RNA was extracted by TRIZOL reagent (Sigma-Aldrich) or by using RNeasy Mini Kit (Qiagen) according to manufacturer’s instructions. cDNA synthesis was obtained by using PrimeScript Reverse Transcriptase (TAKARA, Saint-Germain-en-Laye, France) following manufacturer’s instructions. Quantitative real-time polymerase chain reaction (RT-qPCR) was performed based on the incorporation of SYBR green (Life Technologies). All primers used are listed in the Supplementary Table S7.

**Statistics**

Statistical analyses were performed using GraphPad Prism8 software. All statistical analyses were evaluated with Student’s t-tests and the Mann-Whitney U-test or one-way ANOVA was, as appropriate. A *P* value of < 0.05 was considered statistically significant, where experiments were repeated at least two and three times in triplicates, respectively.

**References**

1. Babaei-Jadidi R, Li N, Saadeddin A, Spencer-Dene B, Jandke A, Muhammad B, et al. FBXW7 influences murine intestinal homeostasis and cancer, targeting Notch, Jun, and DEK for degradation. J Exp Med. 2011;208(2):295-312.

2. O'Rourke KP, Ackerman S, Dow LE, Lowe SW. Isolation, Culture, and Maintenance of Mouse Intestinal Stem Cells. Bio Protoc. 2016;6(4):e1733.

3. Kashfi SMH, Almozyan S, Jinks N, Koo BK, Nateri AS. Morphological alterations of cultured human colorectal matched tumour and healthy organoids. Oncotarget. 2018;9(12):10572-84.

4. Lorenzi F, Babaei-Jadidi R, Sheard J, Spencer-Dene B, Nateri AS. Fbxw7-associated drug resistance is reversed by induction of terminal differentiation in murine intestinal organoid culture. Mol Ther Methods Clin Dev. 2016;3:16024.

5. Talhouni S, Fadhil W, Mongan NP, Field L, Hunter K, Makhsous S, et al. Activated tissue resident memory T-cells (CD8+CD103+CD39+) uniquely predict survival in left sided "immune-hot" colorectal cancers. Front Immunol. 2023;14:1057292.

6. Fujii E, Yamazaki M, Kawai S, Ohtani Y, Watanabe T, Kato A, et al. A simple method for histopathological evaluation of organoids. J Toxicol Pathol. 2018;31(1):81-5.

7. Nateri AS, Spencer-Dene B, Behrens A. Interaction of phosphorylated c-Jun with TCF4 regulates intestinal cancer development. Nature. 2005;437(7056):281-5.

8. Li N, Babaei-Jadidi R, Lorenzi F, Spencer-Dene B, Clarke P, Domingo E, et al. An FBXW7-ZEB2 axis links EMT and tumour microenvironment to promote colorectal cancer stem cells and chemoresistance. Oncogenesis. 2019;8(3):13.

9. Braitbard O, Bishara-Shieban J, Glickstein H, Kott-Gutkowski M, Pace U, Rund DG, et al. An ELISA-based procedure for assaying proteins in digests of human leukocytes and cell lines, using specifically selected peptides and appropriate antibodies. Proteome Sci. 2006;4:14.

**Supplementary Figures Legends**


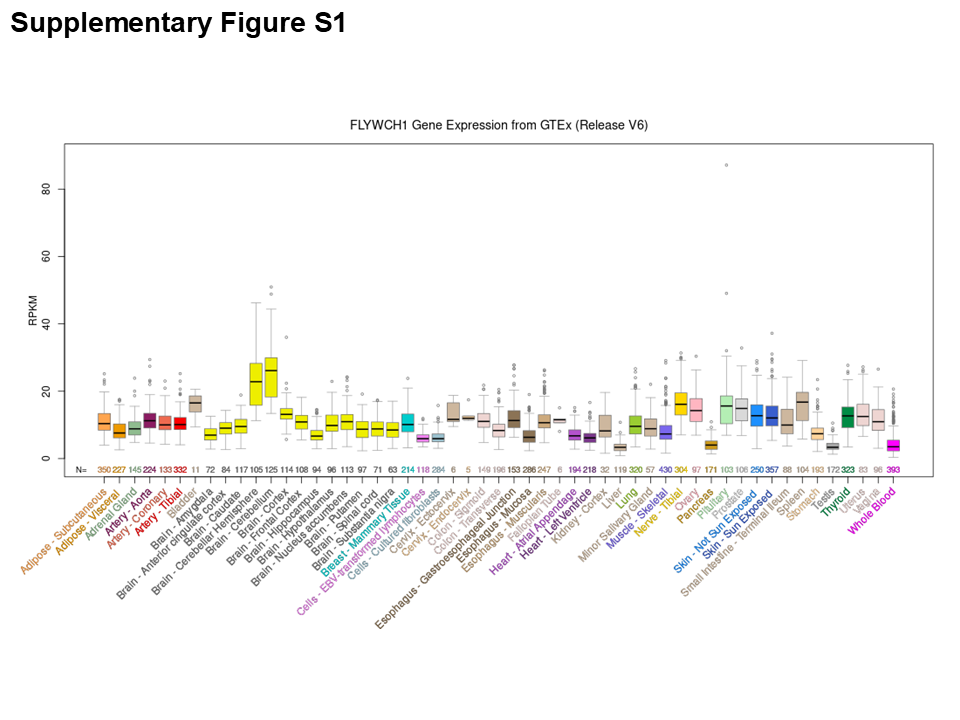


**Supplementary Figure S1.** **FLYWCH1 Tissue Gene Expression from GTEx Extracted from UCSC Genome Browser.**

The gene expression profile of *FLYWCH1* was analysed across 53 tissues using RNA-seq data from 8,555 samples in the GTEx project. The highest median expression was observed in the Brain-Cerebellum at 26.13 RPKM, while the lowest expression was recorded in the Liver at 3.4 RPKM.


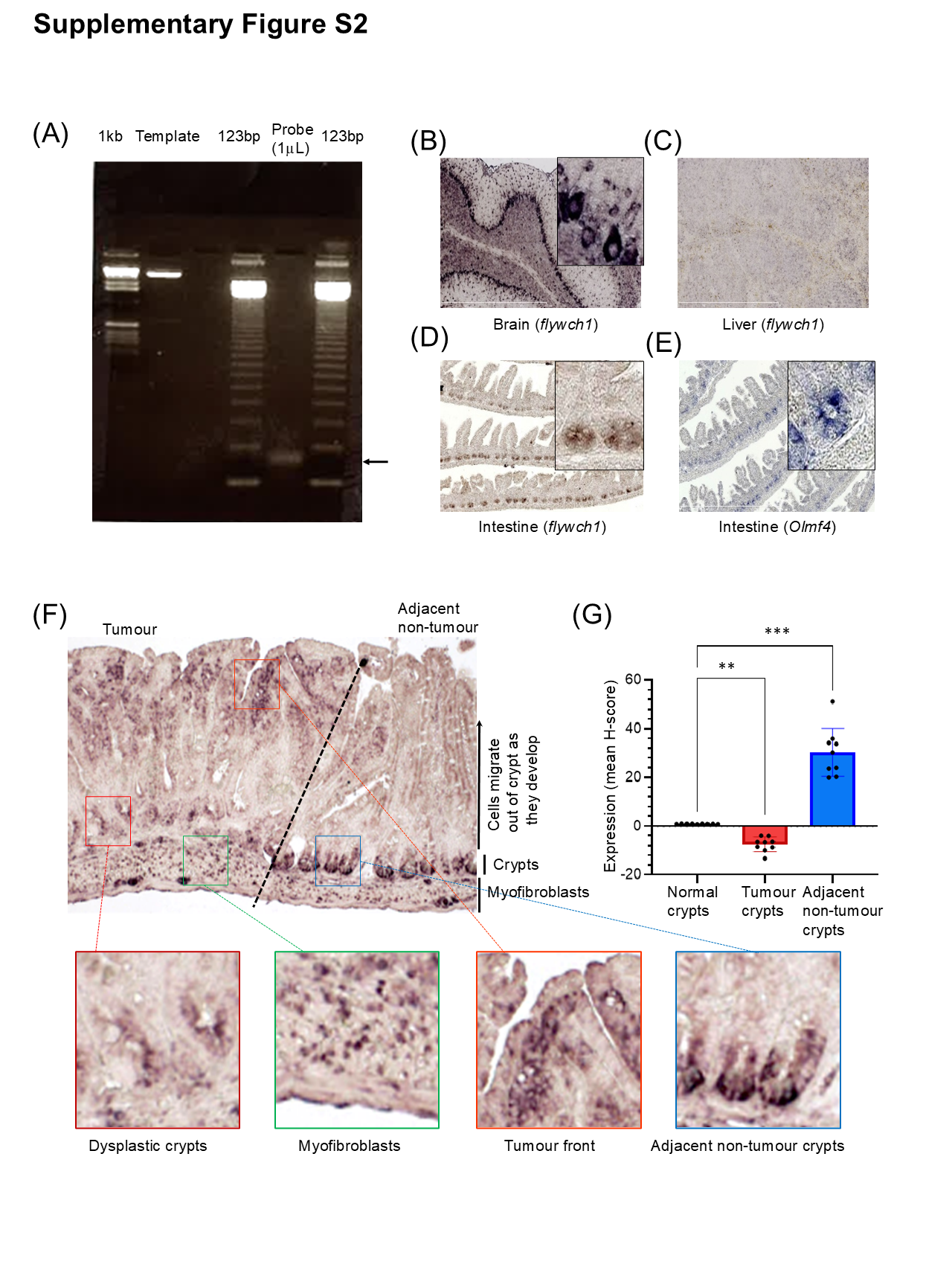


**Supplementary Figure S2. *Flywch1* mRNA Probe and Representative ISH Images.**

(A) Preparation of *Flywch1* mRNA probe: A 651-bp fragment of *Flywch1* cDNA, conserved between mouse and human, spanning three exons of the human *FLYWCH1* gene (exons 1-3), was excised from the eGFP-FLYWCH1-WT clone using *Bam*HI and *Xho*I restriction enzymes. This DNA fragment was subsequently cloned into the multiple cloning site (MCS) of the pcDNA3 vector downstream of the T7 promoter, utilizing the same *Bam*HI and *Xho*I restriction enzymes. To validate the probe's specificity, a nucleotide sequence BLAST search was performed against human and mouse genomic and transcript databases using the NCBI BLAST site (http://blast.ncbi.nlm.nih.gov/Blast.cgi). *In vitro* transcription of this DNA fragment was conducted using T7 RNA polymerase and the digoxigenin (DIG)-RNA labelling mix from Roche (11277073910) was used to generate a human FLYWCH1 RNA probe (lane 4). Lane 1: 1kb ladder, lane 2: pCDNA-*flywch1* template, and lanes 3 and 5: 123-bp DNA ladder. (B-F) Representative ISH Images: Panels (B-E) display brain, liver and intestinal sections from 16-week-old wild-type mice, while panel (F) shows sections from *Apc*^Min^ mice. Panel (B) presents a representative brain section, while panel (C) shows a liver section, labelled with the Flywch1 probe, serving as a low-expression or negative control. Panels (D) and (E) feature *Flywch*1- and *Olfm4*-labeled probes, respectively, with representative images of normal intestinal tissues. Two separate sense and antisense *Flywch1* probes produced consistent results. Scale bar: 100 µm. In *Apc*^Min^ mice, panel (F) illustrates an intestinal tumor along with adjacent non-tumor tissues labelled with the antisense *Flywch1* probe. The boxed areas highlight magnified views of tumor crypts (red), sub-epithelial intestinal myofibroblasts (green), the leading edge of the intestinal tumor (yellow), and normal-appearing crypts adjacent to the tumor (blue). Panel (G) provides *Flywch1*-ISH quantifications of normal, tumor, and non-tumor adjacent intestinal tissues from three separate litters. For the tumor sections, the p-value is 0.008 and for the adjacent non-tumor crypt sections, the p-value is < 0.001.

**
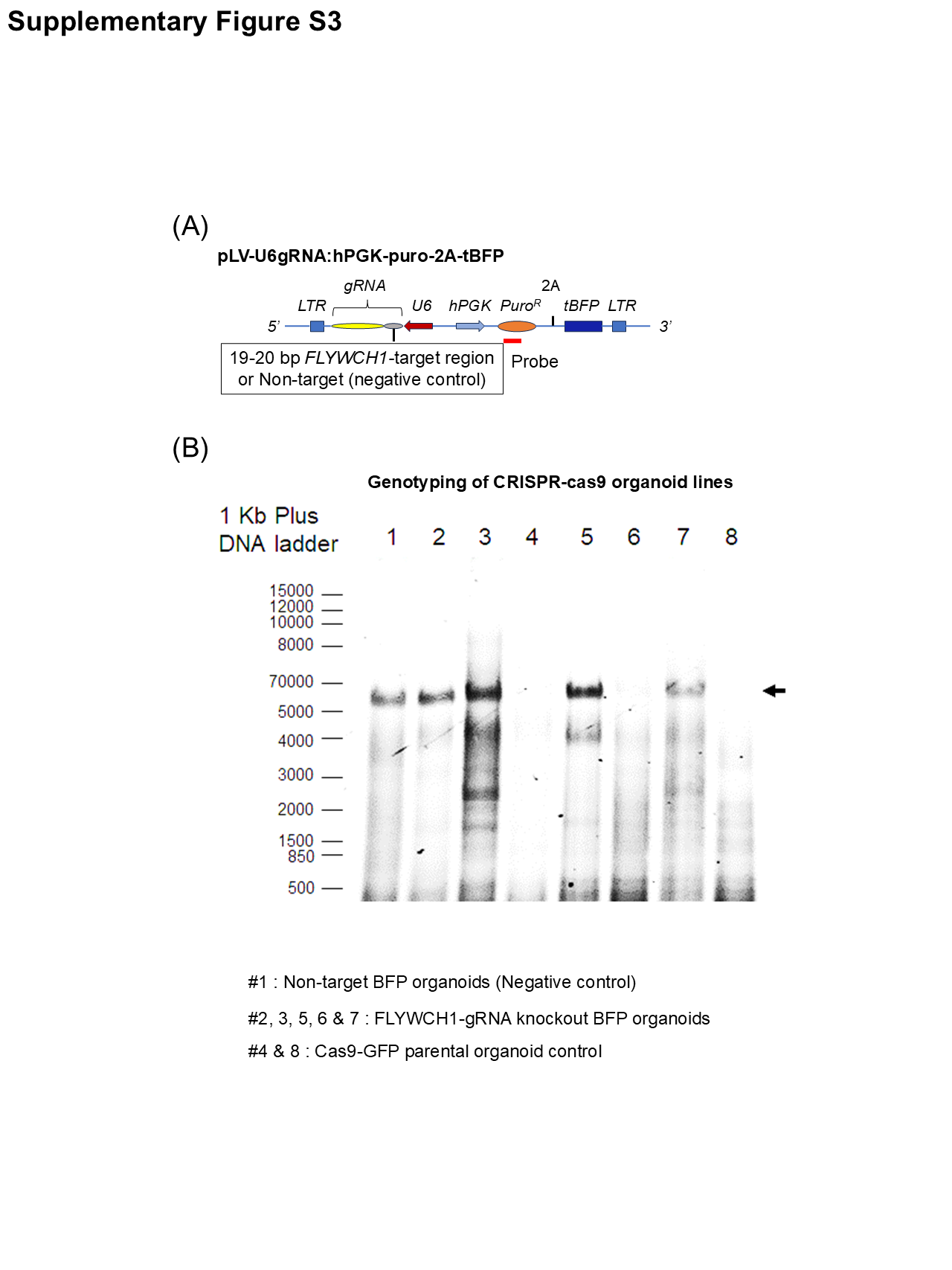
**

**Supplementary Figure S3.** **Validation of Flywch1 Knockout in Organoids via Southern Blotting.**

(A) Schematic presentation of the pLV-U6gRNA lentivirus vector used to express the *Flywch1*-gRNA to knockout the *Flywch1* gene via the CRISPR-Cas9 gene editing system. (B) Representative Southern blot analysis of genomic DNAs: lane 1 shows the non-targeted gRNA organoid line as the 1^st^ negative control; lanes 4 and 8 show two parental organoid lines expressing Cas9 as the 2^nd^ negative control; lanes 2, 3, 5, 6, and 7 show five *Flywch1*-targeted gRNAs lines demonstrating the absence of the *Flywch1* gene in organoids.


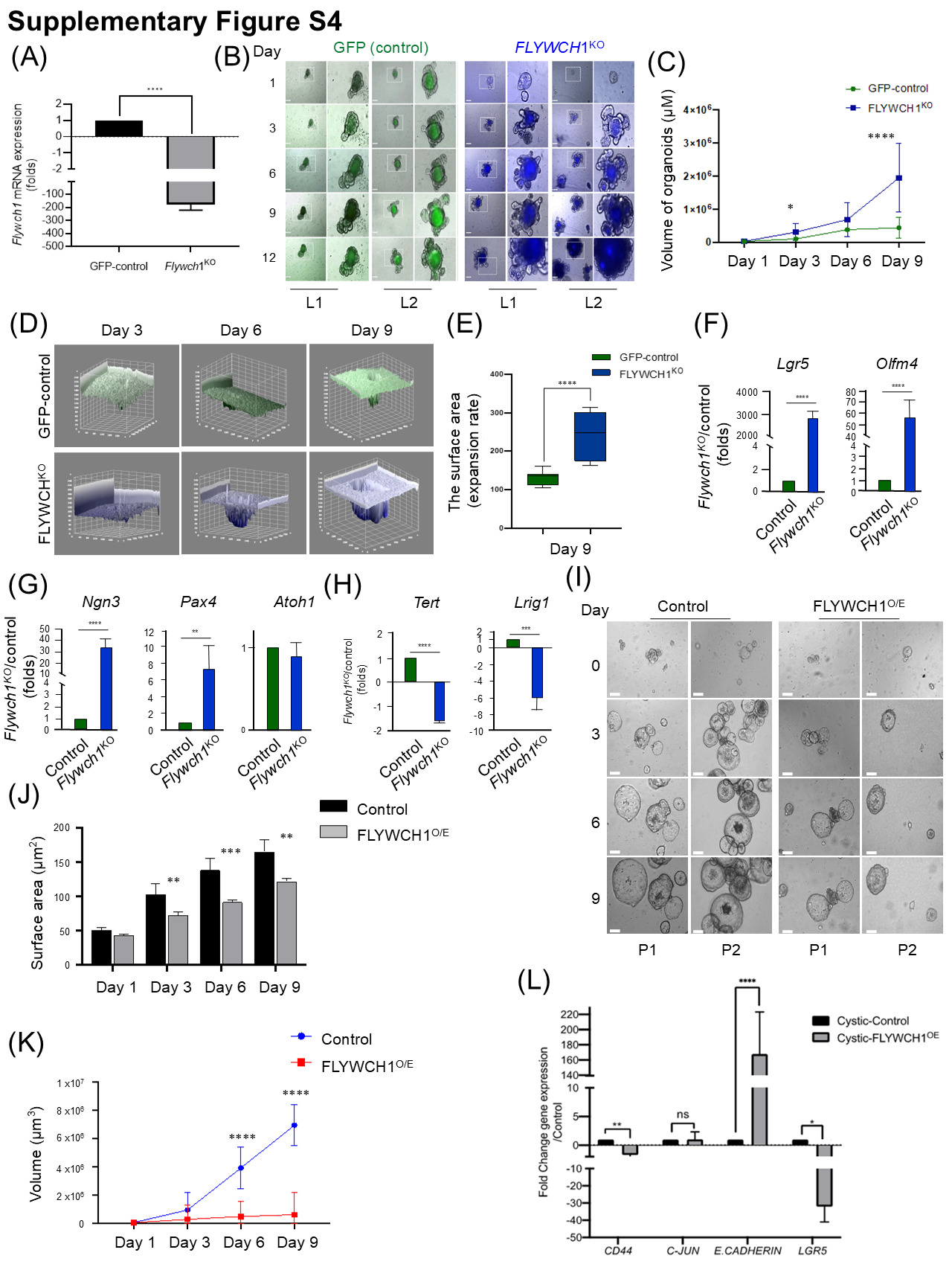


**Supplementary Figure S4. FLYWCH1 Expression is Essential for Maintaining the ISC Pool in Murine Organoids and Tumor PDOs Growth.**

(A) RT-qPCR analysis: Confirmation of *Flywch1* mRNA depletion in murine organoids (2^-(∆∆Ct)). Data represent mean ± SD from three independent lines (N=3), with a significant p-value of < 0.001. (B) Morphological analysis: Representative images show changes in growth patterns between GFP-control and *Flywch1*^KO^ organoids at 2.5x and 10x magnifications. Scale bar: 7.5μm. (C) Organoid volume measurement: The volume of over 35 organoids was measured every two days over the culture time, using the formula; 𝑉 = 4/3𝜋𝑟³. Mean and SD were calculated. P-values for day 3, day 6, and day 9 were 0.01, 0.071, and 0.0001, respectively. (D) 3D interactive surface plot: A representative 3D plot shows morphological differences between GFP-control vs *Flywch1*^KO^ organoids on the indicated days. (E) Surface plot analysis: Using Image-J 3D surface plot plugins, the surface area of 20 organoids was analysed on day 9. Mean ± SD was calculated, with p-values of 0.015 (day 3), 0.071 (day 6), and < 0.001 (day 9). (F) Gene expression analysis - cycling ISCs: *Flywch1*^KO^ upregulated the expression of *Lgr5*+ (*p* < 0.001) and *Olmf4*+ (*p* < 0.001) genes in cycling ISCs. (G) Gene expression analysis - progenitor cells: *Flywch1*^KO^ upregulated *Ngn3* (*p* < 0.001) and *Pax* (*p* = 0.008) progenitor cells, while *Atoh1* expression remains unchanged (*p* = 0.913). (H) Gene expression analysis - quiescent ISCs: *Flywch1*^KO^ downregulated*Lrig1*+ (*p* < 0.001) and *Tert*+ (*p* < 0.001) in quiescence ISCs. mRNA changes were determined using the (2^-(∆∆Ct)) method. and data represent mean and StD. Experiments were carried out in triplicates and repeated on three independent occasions. (I) Morphological analysis of tumor PDOs: Comparison of PDOs overexpressing FLYWCH1 (FLYWCH1^OE^) versus control PDOs in two independent lines. (J) PDO area measurement: The area of over 25 PDOs was measured every two days during culture (Area = πr²). Mean ± SD was calculated with p-values of 0.414 (day 1), 0.002 (day 3), 0.002 (day 6), and < 0.001 (day 9). (K) PDO volume measurement: Volume of over 25 PDOs was measured every two days (Volume = 4/3𝜋𝑟³). Mean ± SD was calculated with p-values of 0.187 (day 1), 0.118 (day 3), < 0.001 (day 6), and < 0.001 (day 9). (L) RT-qPCR analysis of stemness and Wnt target genes: FLYWCH1^OE^ altered the expression of stemness and Wnt target genes in PDOs, including CD44 (*p* = 0.007), C-JUN (*p* = 0.060), E-CADHERIN (*p* = 0.060), and LGR5 (*p* < 0.001). mRNA expression was determined using the 2^-(∆∆Ct) method, N=3. Data represent mean ± SD (* *p* ≤ 0.05, ** *p* ≤ 0.01, *** *p* ≤ 0.001). Experiments were conducted in triplicate and repeated on three independent occasions.


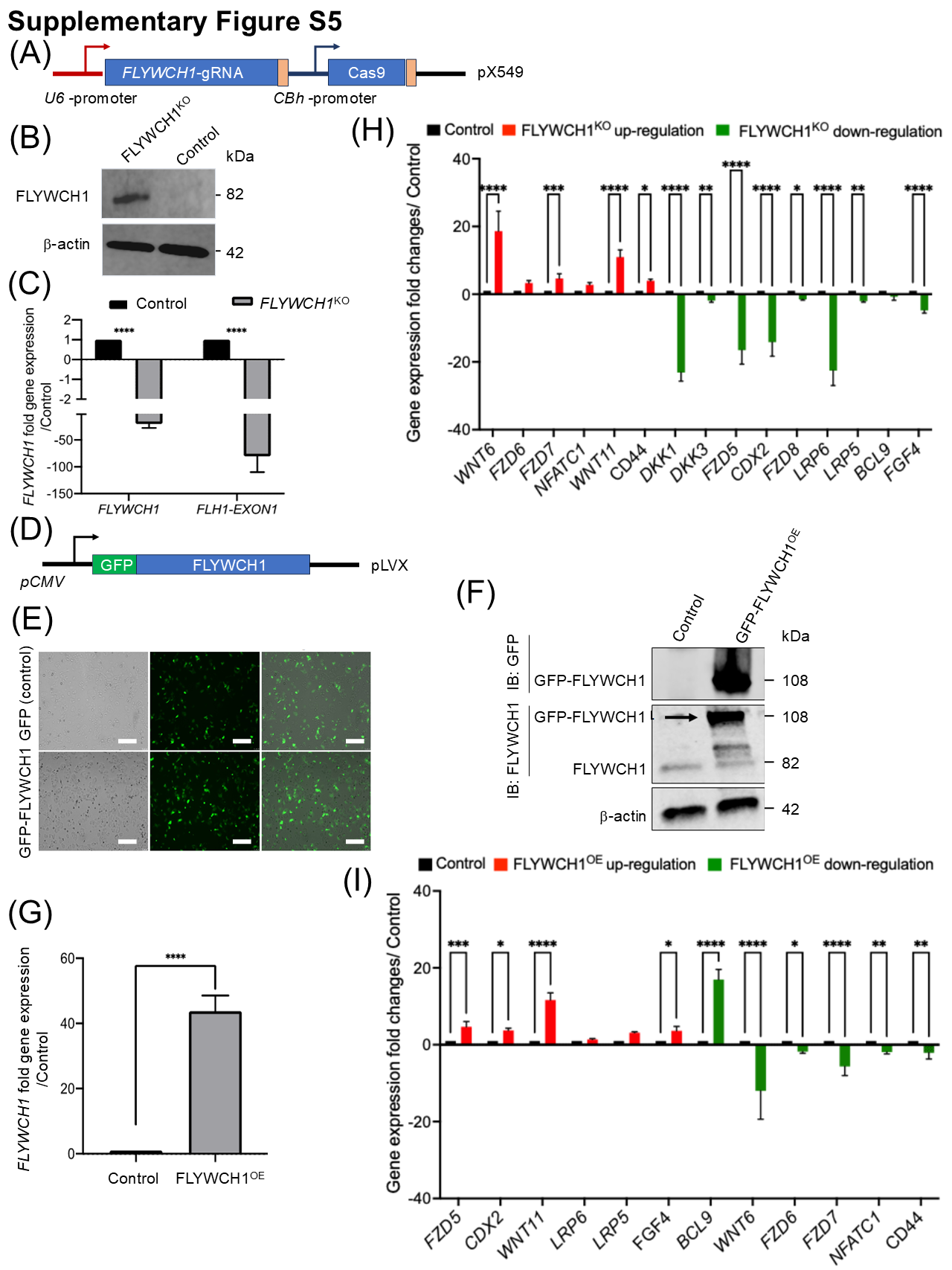


**Supplementary Figure S5. FLYWCH1 Involved in Regulation of Wnt-target Genes Expression in CRC Cells.**

(A) Schematic representation of the pX549-FLYWCH1gRNA-Cas9 construct used to generate FLYWCH1 knockout (*FLYWCH1*^KO^) SW620 cells. The FLYWCH1-gRNA was designed to target exon 1 of the FLYWCH1 gene and was cloned into the pX459-Cas9 plasmid backbone from Addgene. (B) Western blot (WB) analysis using an anti-FLYWCH1 antibody, and (C) qRT-PCR analysis confirming the depletion of FLYWCH1 in *FLYWCH1*^KO^ SW620 cells. Data are presented as mean ± SD (****p* < 0.001). (D) The graph shows the expression of selected Wnt-associated genes affected by the loss of FLYWCH1 expression. Downregulated genes are shown in green, and upregulated genes in red. mRNA expression was assessed using the 2^-(ΔΔCt) method. Data are presented as mean ± SD, with p-values estimated using a two-way ANOVA test (**p* ≤ 0.05, ****p* ≤ 0.001). (E) Schematic representation of the pLVX-Puro-eGFP-FLYWCH1 construct used to generate FLYWCH1 overexpression (FLYWCH1^OE^) SW620 cells. (F) Fluorescent images of GFP showing the lentiviral (LV) transduction efficiency in cells. (G) WB analysis using anti-GFP and anti-FLYWCH1 antibodies, and (H) qRT-PCR analysis validating FLYWCH1 overexpression in cells. (I) The graph shows the expression of selected Wnt-associated genes affected by FLYWCH1 overexpression. Downregulated genes are shown in green, and upregulated genes in red. mRNA expression was assessed using the 2^-(ΔΔCt) method. Data are presented as mean ± SD, with p-values estimated using a two-way ANOVA test (**p* ≤ 0.05, ***p* ≤ 0.01, ****p* ≤ 0.001).


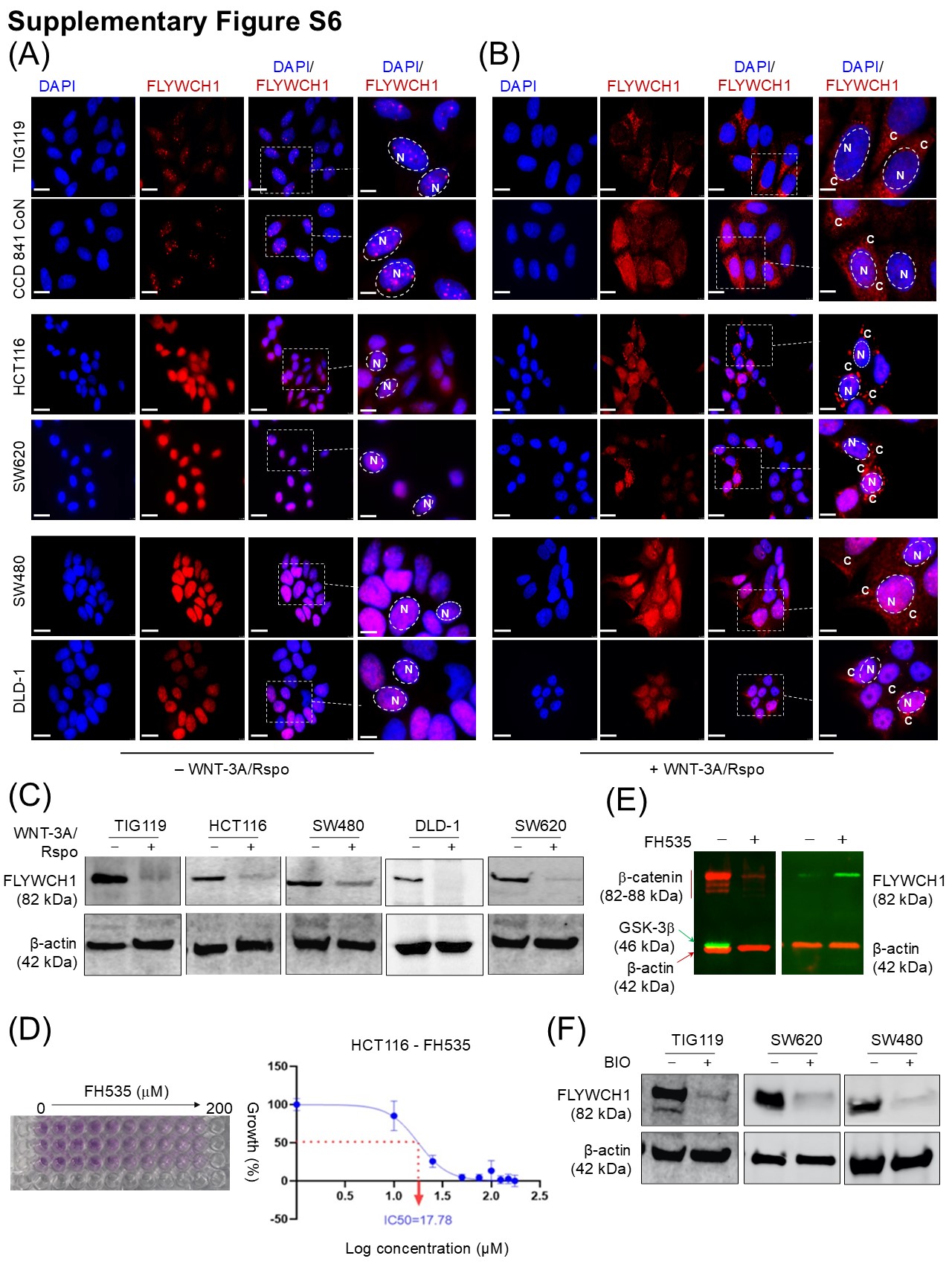


**Supplementary Figure S6. Effects of Wnt Activation and Small Molecule Compounds (FH535 & BIO) on Cellular Distribution and Expression Levels of FLYWCH1 Protein.**

(A, B) Immunofluorescence staining shows the effects of Wnt-3A/Rspo on the distribution and expression pattern of FLYWCH1 in normal and cancer cell lines. Dotted lines indicate enlarged cells. Magnification: 100x. Scale bars for the original images: 7.5 μm. Scale bars for the zoomed-in images: 50 μm. (C) Immunoblot analysis reveals a decrease in total FLYWCH1 protein levels in TIG119 and CRC cells following Wnt-3A/Rspo treatment. (D) The SRB colorimetric survival assay, performed in triplicate on synchronized serum-starved HCT116 cells, shows the response to 10 increasing concentrations of FH535. The IC50 value (17.78 µmol/L) was calculated using GraphPad Prism software 7.02 and represents the mean of three independent experiments ± SEM, with *p* ≤ 0.005. (E) FH535, a nuclear β-catenin inhibitor, increases FLYWCH1 expression while reducing GSK-3β protein levels in CRC cell lines. The left panel shows β-Catenin (red) and GSK-3β (green), while the right panel shows FLYWCH1 (green) with β-actin (red) as the loading control. The immunoblot experiment was repeated at least twice independently. (F) Immunoblot analysis shows a reduction in total FLYWCH1 protein levels in TIG119 and CRC cells following treatment with the GSK-3β phosphorylation inhibitor BIO. The immunoblot experiments were repeated at least three times independently.

**
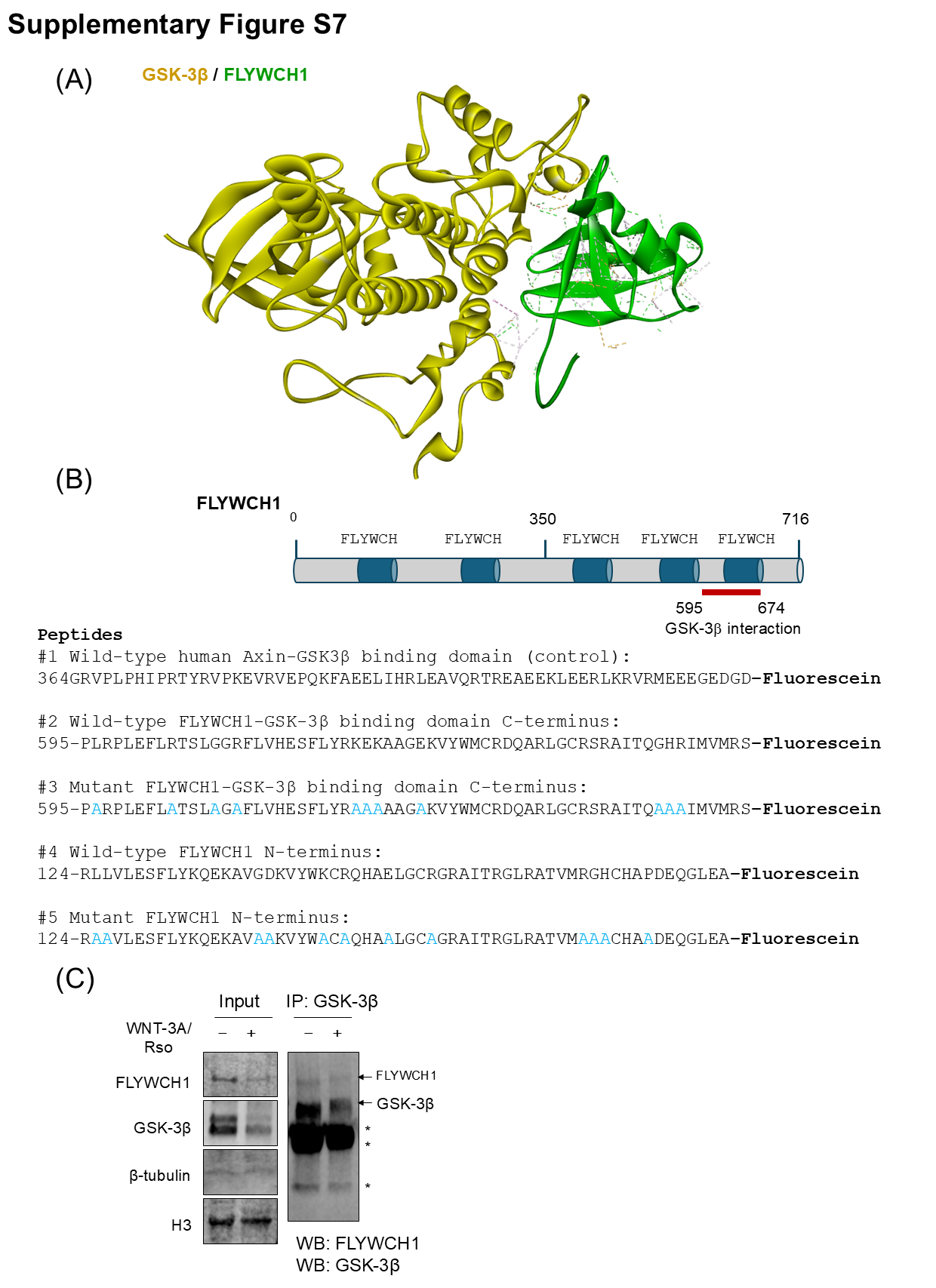
**

**Supplementary Figure S7. Protein-protein Interaction Model, Binding Peptide Sequences, and Co-immunoprecipitation Analysis of FLYWCH1 and GSK-3β.**

(A) The protein-protein interaction model 3 between FLYWCH1 (Green- 2RPR) and GSK-3β (1GNG) demonstrates the lowest binding energy (ΔG = -12.7 kcal/mol), as shown in Supplementary Table S3. (B) The interaction between FLYWCH1 and GSK-3β occurs within the amino acid residues 595 to 674 (<https://www.rcsb.org/sequence/2rpr>), highlighted in red. The sequences of the wild-type C-terminus (#2), mutant peptide (#3), and wild-type N-terminus (#4) of FLYWCH1, along with the wild-type peptide from the Axin-GSK-3β interaction binding domain (#1), were used for ELISA-based binding assays with the GSK-3β antibody (Figure 1F). (C) Co-Immunoprecipitation (Co-IP) analysis revealed that Wnt-3A/Rspo treatment does not affect the nuclear interaction between FLYWCH1 and GSK-3β in HEK293T cells. Cells were treated or mock-treated with Wnt-3A/Rspo, and after 24 hours, nuclear fractions and total protein were extracted and subjected to immunoprecipitation (IP) using an antibody against endogenous GSK-3β (with beads only serving as a negative control). The complexes and input samples were subsequently immunoblotted with the FLYWCH1 antibody as indicated. Co-IP and subsequent immunoblot experiments were performed in at least three independent experiments.


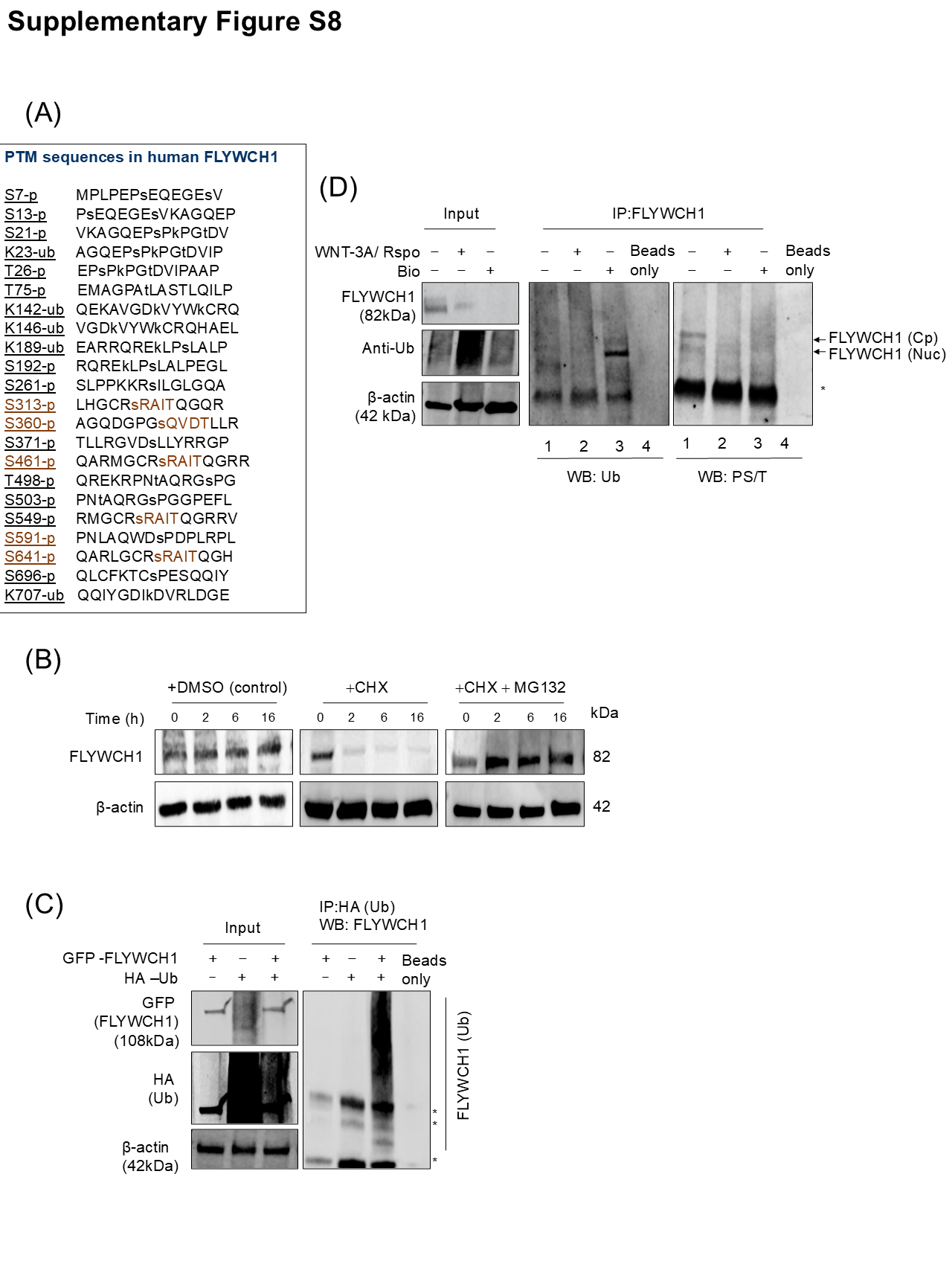


**Supplementary Figure S8. Post-translational Modifications and Stability of Human FLYWCH1 Protein.**

(A) Amino acid sequences showing the post-translational modification (PTM) for the human FLYWCH1 protein. The GSK-3β consensus phosphorylation sequences, S/Txxxp(S/T), within the FLYWCH1 protein are highlighted in red. (B) CHX-chase analysis demonstrating the half-life of the FLYWCH1 protein. HEK293T cells were treated with DMSO (left panel), 100 μg/ml CHX (middle panel), or 100 μg/ml CHX + 2 μmol/L MG132 (right panel). Lysates were collected at the indicated time points, and 80 μg of protein was subjected to 4-20% SDS-PAGE followed by immunoblotting with anti-FLYWCH1 and anti-β-actin antibodies. (C) *In vitro* ubiquitination assay demonstrating that FLYWCH1 can be modified by ubiquitin (UB). HEK293T cells were transiently transfected with GFP-FLYWCH1 and/or HA-Ub. Forty-eight hours post-transfection, lysates were prepared and immunoprecipitated with an HA-tagged antibody, followed by immunoblotting with anti-FLYWCH1. The left panel shows the characteristic smear of ubiquitin-conjugated FLYWCH1. (D) Co-immunoprecipitation (Co-IP) assay showing the effects of Wnt-3A/Rspo and BIO on the phosphorylation and ubiquitination of FLYWCH1. HEK293T cells were treated or mock-treated for 24 hours. Lysates were then immunoprecipitated for endogenous FLYWCH1 and blotted with anti-Phospho-(Ser/Thr) or anti-UB antibodies. Beads only served as a negative control for the IP, and β-actin was used as a loading control. All Co-IP, ubiquitination, and subsequent immunoblotting experiments were repeated at least three times independently.


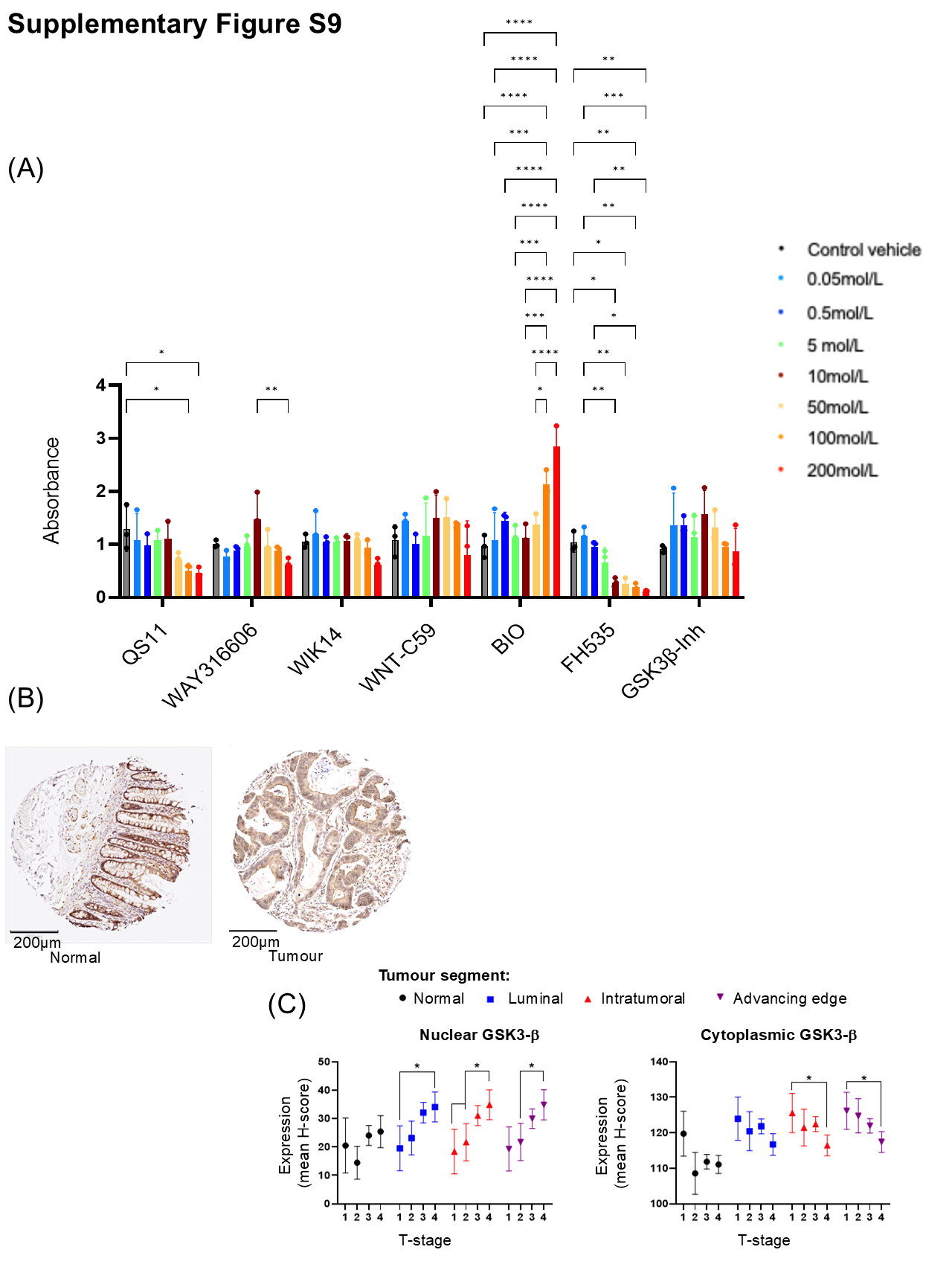


**Supplementary Figure S9. Clinical Relevance of FLYWCH1/GSK-3β Expression in CRC Patients.**

(A) Six compounds–QS11, WAY-316606, WIK14, BIO, FH535, and a GSK-3β inhibitor–were tested at eight concentrations ranging from 0.05 to 200 mmol/L, and cell viability was assessed using the WST-1 assay. Notably, BIO treatment promoted the proliferation of patient-derived organoids (PDOs), while FH535 consistently inhibited their growth. Tumor organoid medium served as the blank control. The WST-1 assay was performed in triplicate, with the experiment repeated twice independently. Statistical significance was evaluated using a two-way ANOVA test (* *p* ≤ 0.05, ** *p* ≤ 0.01, *** *p* ≤ 0.001). The p-values for statistically significant drug effects are as follows:

**QS11:**

Control vehicle vs. 100 μmol/L, *p* = 0.022

Control vehicle vs. 200 μmol/L, *p* = 0.010

**BIO:**

Control vehicle vs. 100 μmol/L, *p* < 0.001

Control vehicle vs. 200 μmol/L, *p* < 0.001

0.05 μmol/L vs. 100 μmol/L, *p* < 0.001

0.05 μmol/L vs. 200 μmol/L, *p* < 0.001

0.5 μmol/L vs. 200 μmol/L, *p* < 0.001

5 μmol/L vs. 100 μmol/L, *p* < 0.001

5 μmol/L vs. 200 μmol/L, *p* < 0.001

10 μmol/L vs. 100 μmol/L, *p* < 0.001

10 μmol/L vs. 200 μmol/L, *p* < 0.001

50 μmol/L vs. 100 μmol/L, *p* = 0.026

50 μmol/L vs. 200 μmol/L, *p* < 0.001

**FH535:**

Control vehicle vs. 10 μmol/L, *p* = 0.031

Control vehicle vs. 50 μmol/L, *p* = 0.019

Control vehicle vs. 100 μmol/L, *p* = 0.009

Control vehicle vs. 200 μmol/L, *p* = 0.003

0.05μmol/L vs. 10 μmol/L, *p* = 0.006

0.05μmol/L vs. 50 μmol/L, *p* = 0.004

0.05μmol/L vs. 100 μmol/L, *p* = 0.002

0.05μmol/L vs. 200 μmol/L, *p* < 0.001

0.5μmol/L vs. 100 μmol/L, *p* = 0.027

0.5μmol/L vs. 200 μmol/L, *p* = 0.009.

(B) Representative images of IHC staining for GSK-3β in CRC TMA. The IHC analysis shows varying intensities of nuclear and cytoplasmic staining between normal tissues (left panel) and tumor tissues (right panel). Scale bar: 200 μm. (C) Mean nuclear (left panel) and cytoplasmic (right panel) expression levels of GSK-3β across different tumor segments and pathological T stages. The stages T1, T2, T3, and T4 are represented by 1, 2, 3, and 4, respectively. Detailed information on the number of samples, 95% confidence intervals, and p-values is provided in Supplementary Table S5.

**
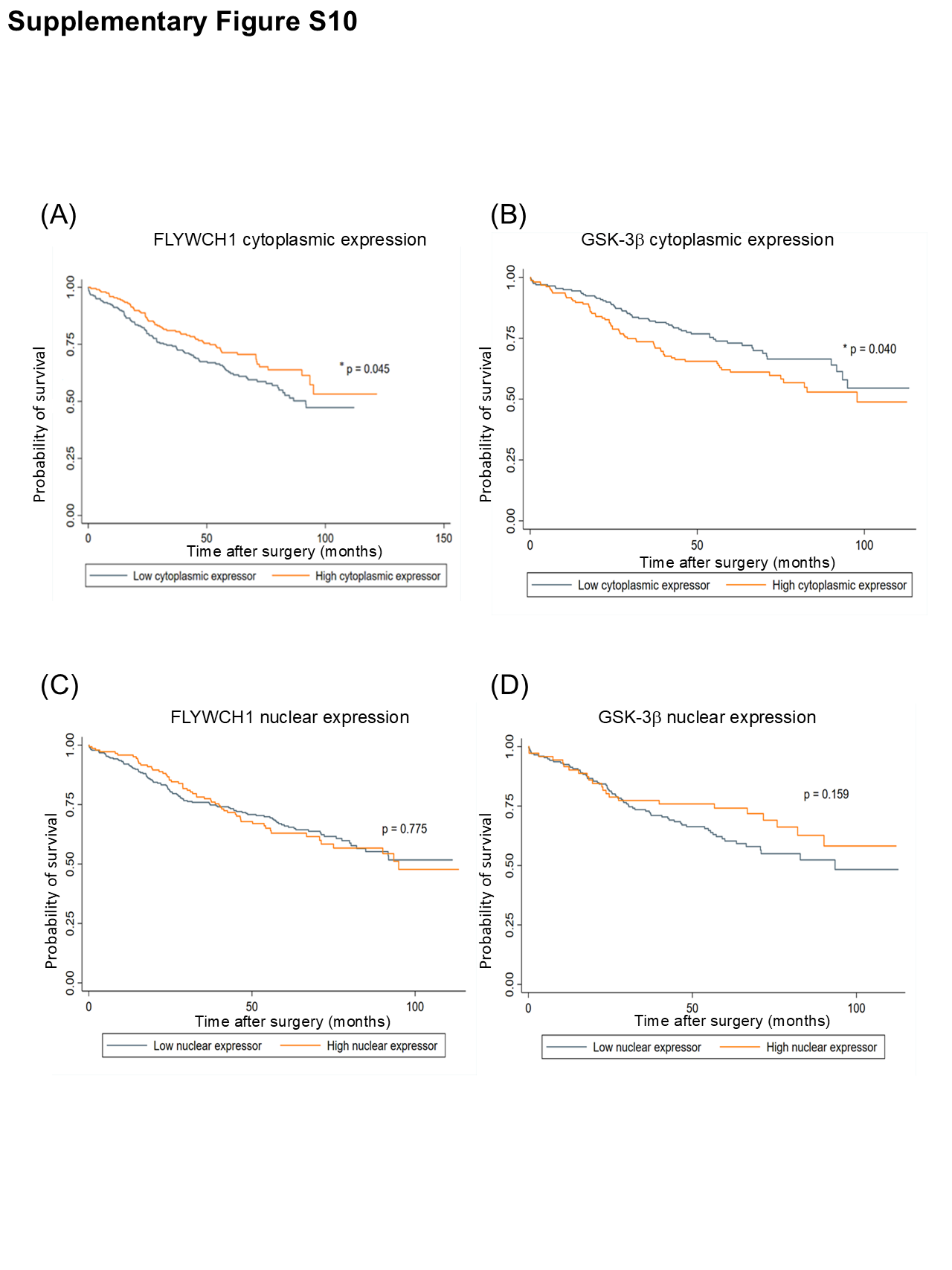
**

**Supplementary Figure S10. Kaplan-Meier Analysis of Overall Survival Based on High and Low Tumor Expression of FLYWCH1 and GSK-3β in Both the Nucleus and Cytoplasm.**

(A) Comparison of overall survival between high and low cytoplasmic expression of FLYWCH1. (B) Comparison of overall survival between high and low cytoplasmic expression of GSK-3β. (C) Comparison of overall survival between high and low nuclear expression of FLYWCH1. (D) Comparison of overall survival between high and low nuclear expression of GSK-3β.

Survival outcomes: For FLYWCH1, low expressors have a mean survival time of 74.9 months, whereas high expressors have a mean survival time of 88.3 months. Hazard Ratio (HR): 0.72 (95% CI: 0.53 – 0.99), n=241/201 (**p* ≤ 0.05). For GSK-3β, low expressors have a mean survival time of 85.1 months, while high expressors have a mean survival time of 75.7 months. HR: 1.45 (95% CI: 1.01 – 2.06), n=199/158 (**p* ≤ 0.05). Detailed data including the number of tissues analysed, 95% confidence intervals, and p-values are provided in Supplementary Tables S1, S5, and S6.

**Supplementary Video S1. The video demonstrates the protein-protein docking interactions between FLYWCH1 (Green) and GSK-3β.**

**Supplementary Tables**

**Supplementary Table S1. Detailed information on immunohistochemistry data and statistical analysis, including the applied statistical method, 95% confidence interval (CI), and p-values, for FLYWCH1 staining across various tumor segments and stages in TMA slides.**

| **Tumor segments** | **Number** | **Mean** | **95CI (low)** | **95CI (high)** | **SD** |
| --- | --- | --- | --- | --- | --- |
| **FLYWCH1 - Nuclear (Fig. 1B, left panel and S10C)** | | | | | |
| Normal | 596 | 155.88 | 152.52 | 159.24 | 41.78 |
| Luminal | 667 | 86.7 | 84.48 | 88.94 | 29.38 |
| Intratumoral | 734 | 85.23 | 83 | 86.46 | 30.78 |
| Advancing Edge | 753 | 90.64 | 88.56 | 92.73 | 29.1 |
| **One-way ANOVA** | | | | | |
| **Norm vs Lum: Diff=-69.2800, 95%CI=-74.0230 to -64.5370, *p*=0.0000** | | | | | |
| **Norm vs Tum: Diff=-70.7500, 95%CI=-75.3897 to -66.1103, *p*=0.0000** | | | | | |
| **Norm vs Ae: Diff=-65.3400, 95%CI=-69.9534 to -60.7266, *p*=0.0000** | | | | | |
| Lum vs Tum: Diff=-1.4700, 95%CI=-5.9713 to 3.0313, *p*=0.8358 | | | | | |
| Lum vs Ae: Diff=3.9400, 95%CI=-0.5342 to 8.4142, *p*=0.1070 | | | | | |
| **Tum vs Ae: Diff=5.4100, 95%CI=1.0454 to 9.7746, *p*=0.0079** | | | | | |
| **FLYWCH1 - Cytoplasmic (Fig. 1B, right panel and S10A)** | | | | | |
| Normal | 598 | 122.22 | 119.83 | 124.62 | 29.81 |
| Luminal | 686 | 131.8 | 129.28 | 134.32 | 33.6 |
| Intratumoral | 753 | 132.834 | 130.57 | 135.1 | 31.66 |
| Advancing Edge | 773 | 140.71 | 138.58 | 142.83 | 30.12 |
| **Tumor stages** | **Number** | **Mean** | **95CI (low)** | **95CI (high)** | **SD** |
| Normal, T1 | 47 | 125.43 | 114.88 | 135.96 | 35.89 |
| Normal, T2 | 59 | 127.54 | 120.25 | 134.84 | 27.98 |
| Normal, T3 | 322 | 121.52 | 118.28 | 124.76 | 29.55 |
| Normal, T4 | 170 | 120.82 | 116.42 | 125.22 | 29.06 |
| Luminal, T1 | 55 | 140.45 | 133.42 | 147.49 | 26.04 |
| Luminal, T2 | 75 | 137.4 | 130.14 | 144.66 | 31.55 |
| Luminal, T3 | 359 | 130.96 | 127.41 | 134.52 | 34.24 |
| Luminal, T4 | 197 | 128.78 | 123.92 | 133.65 | 34.61 |
| Intratumoral, T1 | 62 | 134.68 | 125.5 | 143.86 | 36.16 |
| Intratumoral, T2 | 82 | 141.71 | 134.97 | 148.45 | 30.67 |
| Intratumoral, T3 | 390 | 133.53 | 130.4 | 136.65 | 31.41 |
| Intratumoral, T4 | 219 | 127.76 | 123.71 | 131.81 | 30.4 |
| Advancing Edge, T1 | 63 | 146.83 | 138.72 | 154.93 | 32.18 |
| Advancing Edge, T2 | 86 | 150.29 | 144.58 | 156 | 26.62 |
| Advancing Edge, T3 | 404 | 140.45 | 137.43 | 146.46 | 30.82 |
| Advancing Edge, T4 | 220 | 135.68 | 131.9 | 139.46 | 28.43 |
| **One-way ANOVA - Intratumoral group & Tukey Honest Significant Difference (HSD) Post-hoc Test.** | | | | | |
| Tum, T1 vs Tum, T2: Diff=7.0300, 95%CI=-6.5970 to 20.6570, *p*=0.545 | | | | | |
| Tum, T1 vs Tum, T3: Diff=-1.1500, 95%CI=-12.2204 to 9.9204, *p*=0.993 | | | | | |
| Tum, T1 vs Tum, T4: Diff=-6.9200, 95%CI=-18.5682 to 4.7282, *p*=0.420 | | | | | |
| Tum, T2 vs Tum, T3: Diff=-8.1800, 95%CI=-18.0168 to 1.6568, *p*=0.141 | | | | | |
| **Tum, T2 vs Tum, T4: Diff=-13.9500, 95%CI=-24.4328 to -3.4672, *p*=0.0036** | | | | | |
| Tum, T3 vs Tum, T4: Diff=-5.7700, 95%CI=-12.6072 to 1.0672, *p*=0.131 | | | | | |
| AE, T1 vs AE, T2: Diff=3.4600, 95%CI=-9.2717 to 16.1917, *p*=0.897 | | | | | |
| AE, T1 vs AE, T3: Diff=-6.3800, 95%CI=-16.7794 to 4.0194, *p*=0.391 | | | | | |
| **AE, T1 vs AE, T4: Diff=-11.1500, 95%CI=-22.1204 to -0.1796, *p*=0.044** | | | | | |
| **AE, T2 vs AE, T3: Diff=-9.8400, 95%CI=-18.9574 to -0.7226, *p*=0.028** | | | | | |
| **AE, T2 vs AE, T4: Diff=-14.6100, 95%CI=-24.3736 to -4.8464, *p*=0.000** | | | | | |
| AE, T3 vs AE, T4: Diff=-4.7700, 95%CI=-11.2028 to 1.6628, *p*=0.225 | | | | | |

**Supplementary Table S2. The scores of HADDOCK binding for FLYWCH1/GSK-3β (Cluster 1) complex.**

| **Variables (Cluster 1)** | **Scores** |
| --- | --- |
| HADDOCK score | -84.9 +/- 14.6 |
| Cluster size | 10 |
| *RMSD from the overall lowest-energy structure | 1.0 +/- 0.6 |
| Van der Waals energy | -64.7 +/- 13.6 |
| Electrostatic energy | -481.0 +/- 48.9 |
| Desolvation energy | 15.4 +/- 2.5 |
| Restraints violation energy | 605.6 +/- 92.7 |
| Buried Surface Area | 2364.6 +/- 120.9 |
| Z-Score | -1.8 |

*RMSD: Root Mean Square deviation

**Supplementary Table S3. PRODIGY analysis of the lowest binding energy for the top models from cluster 1.**

| **Protein-protein complex** | **ΔG (kcal mol-1)** | **Kd (M) at ℃** | **ICs charged-charged** | **ICs charged-polar** | **ICs charged-apolar** | **ICs polar-polar** | **ICs polar-apolar** | **ICs apolar-apolar** | **NIS charged** |
| --- | --- | --- | --- | --- | --- | --- | --- | --- | --- |
| Model 1 | -11.8 | 2.30E-09 | 16 | 19 | 39 | 5 | 13 | 14 | 28.13 |
| Model 2 | -12.3 | 9.80E-10 | 20 | 15 | 26 | 2 | 16 | 12 | 28.36 |
| ***Model 3** | **-12.7** | **4.90E-10** | **23** | **14** | **26** | **4** | **19** | **7** | **28.15** |
| Model 4 | -11.7 | 2.70E-09 | 17 | 17 | 26 | 4 | 17 | 8 | 28.66 |

*Model 3 has lowest binding energy (ΔG) used for interaction analysis.

**Supplementary Table S4. Model 3 - FLYWCH1/GSK-3β interacting residues and type of bonds.**

| **FLYWCH1** | **GSK-3β** | **Bond** |
| --- | --- | --- |
| Leu 595 | Ile 376 | Alkyl bond |
| Leu 595 | Ala 336 | Alkyl bond |
| Arg 602 | His 299 | Conventional Hydrogen bond |
| Arg 602 | Glu 322 | Salt bridge charge-charge |
| Arg 602 | Glu 322 | Conventional Hydrogen bond |
| Gly 606 | Thr 277 | Conventional Hydrogen bond |
| Arg 608 | Glu 322 | Salt bridge charge-charge |
| Arg 608 | Glu 322 | Conventional Hydrogen bond |
| Arg 608 | Glu 279 | Three Salt bridge charge-charge |
| Arg 608 | Glu 279 | Carbon bond |
| Arg 608 | Glu 279 | Charge-charge |
| Arg 608 | Thr 324 | Two Conventional Hydrogen bonds |
| Arg 608 | Thr 324 | Donor- Donor bond |
| Lys 619 | Glu 283 | Salt bridge charge-charge |
| Ala 622 | Glu 283 | Conventional Hydrogen bond |
| Glu 620 | Arg 283 | Conventional Hydrogen bond |
| Glu 625 | Thr 326 | Carbon bond |
| Gly 647 | Arg 319 | Three Conventional Hydrogen bond |
| His 648 | Ala 336 | Pi-alkyl bond |
| His 648 | Ala 336 | Conventional Hydrogen bond |
| His 648 | His 337 | Pi-Pi staked bond |
| His 648 | Arg 319 | Conventional Hydrogen bond |
| Arg 649 | Glu 333 | Conventional Hydrogen bond |
| Arg 649 | Glu 333 | Salt bridge charge-charge |
| Glu 672 | Lys 297 | Two Conventional Hydrogen bonds |
| Glu 672 | Lys 297 | Charge-charge bond |

**Supplementary Table S5. Detailed information on immunohistochemistry data and statistical analysis, including the applied statistical method, 95% confidence interval (CI), and p-values, for GSK-3β staining across various tumor segments and stages in TMA slides.**

| **Tumor segments** | **Number** | | | **Mean** | **95CI (low)** | **95CI (high)** | **SD** |
| --- | --- | --- | --- | --- | --- | --- | --- |
| **GSK-3β – Nuclear (Fig. S9C, left panel and S10D)** | | | | | | | |
| Normal | 483 | | | 23.05383 | 20.43335 | 25.67431 | 29.30986 |
| Luminal | 579 | | | 30.46632 | 27.88252 | 33.05013 | 31.65488 |
| Intratumoral | 614 | | | 29.87296 | 27.29503 | 32.4509 | 32.52742 |
| Advancing Edge | 604 | | | 29.38742 | 26.84785 | 31.92699 | 31.78032 |
| **Tumor stages** | | | | | | | |
| Norm_T1 | 37 | | | 20.54054 | 10.83748 | 30.2436 | 29.10192 |
| Norm_T2 | 56 | | | 14.46429 | 8.660287 | 20.26828 | 21.67274 |
| Norm_T3 | 260 | | | 24.07692 | 20.54506 | 27.60878 | 28.92064 |
| Norm_T4 | 130 | | | 25.42308 | 19.79387 | 31.05229 | 32.43977 |
| Lum_T1 | 54 | | | 19.53704 | 11.64464 | 27.42943 | 28.91543 |
| Lum_T2 | 65 | | | 23.15385 | 17.2098 | 29.09789 | 23.98843 |
| Lum_T3 | 299 | | | 32.0903 | 28.47043 | 35.71017 | 31.80628 |
| Lum_T4 | 161 | | | 34.06832 | 28.79362 | 39.34303 | 33.88955 |
| Tum_T1 | 59 | | | 18.38983 | 10.55892 | 26.22075 | 30.04939 |
| Tum_T2 | 68 | | | 21.69118 | 15.13991 | 28.24244 | 27.06557 |
| Tum_T3 | 315 | | | 31.06984 | 27.51678 | 34.6229 | 32.05034 |
| Tum_T4 | 172 | | | 34.85465 | 29.61139 | 40.09792 | 34.83641 |
| AE_T1 | 60 | | | 19.33333 | 11.57087 | 27.09579 | 30.04892 |
| AE_T2 | 63 | | | 21.74603 | 15.23358 | 28.25848 | 25.85878 |
| AE_T3 | 317 | | | 29.98423 | 26.54392 | 33.42453 | 31.13237 |
| AE_T4 | 164 | | | 34.84756 | 29.53808 | 40.15705 | 34.43418 |
| **One-way ANOVA, Luminal** | | | | | | | |
| t1 vs. t2: Diff= -3.617, 95%CI= -18.51 to 11.27, *p*= 0.923 | | | | | | | |
| **t1 vs. t3: Diff= -12.55, 95%CI= -24.51 to -0.5966, *p*= 0.035** | | | | | | | |
| **t1 vs. t4: Diff= -14.53, 95%CI= -27.25 to -1.815, *p*= 0.017** | | | | | | | |
| t2 vs. t3: Diff= -8.936, 95%CI= - 20.00 to 2.130, *p*= 0.160 | | | | | | | |
| t2 vs. t4: Diff= -10.91, 95%CI= -22.80 to 0.9689, *p*= 0.084 | | | | | | | |
| t3 vs. t4: Diff=-1.978, 95%CI= -9.883 to 5.927, *p*= 0.917 | | | | | | | |
| **One-way ANOVA, Intratumoral** | | | | | | | |
| t1 vs. t2: Diff= -3.301, 95%CI= -18.05 to 11.45, *p*= 0.939 | | | | | | | |
| **t1 vs. t3: Diff= -12.68, 95%CI= -24.44 to -0.9215, *p*= 0.028** | | | | | | | |
| **t1 vs. t4: Diff= -16.46, 95%CI= -28.97 to -3.959, *p*= 0.004** | | | | | | | |
| t2 vs. t3: Diff= -9.379, 95%CI= -20.46 to 1.705, *p*= 0.130 | | | | | | | |
| **t2 vs. t4: Diff= -13.16, 95%CI= -25.04 to -1.290, *p*= 0.022** | | | | | | | |
| t3 vs. t4: Diff= -3.785, 95%CI= -11.64 to 4.074, *p*= 0.601 | | | | | | | |
| **One-way ANOVA, Advancing Edge** | | | | | | | |
| t1 vs. t2: Diff= -2.413, 95%CI= -17.04 to 12.21, *p*= 0.974 | | | | | | | |
| t1 vs. t3: Diff= -10.65, 95%CI= -22.07 to 0.7640, *p*= 0.077 | | | | | | | |
| **t1 vs. t4: Diff= -15.51, 95%CI= -27.75 to -3.281, *p*= 0.006** | | | | | | | |
| t2 vs. t3: Diff= -8.238, 95%CI= -19.42 to 2.946, *p*= 0.230 | | | | | | | |
| **t2 vs. t4: Diff= -13.1, 95%CI= -25.12 to -1.084, *p*= 0.026** | | | | | | | |
| t3 vs. t4: Diff= -4.863, 95%CI= -12.66 to 2.935, *p*= 0.375 | | | | | | | |
| **GSK-3β – Cytoplasmic (Fig. S9C, right panel and S10B)** | | | | | | | |
| Normal | | 489 | 111.922 | | 110.382 | 113.462 | 17.331 |
| Luminal | | 585 | 120.427 | | 118.818 | 122.036 | 19.810 |
| Intratumoral | | 613 | 121.435 | | 119.384 | 122.538 | 19.171 |
| Advancing Edge | | 624 | 120.961 | | 119.914 | 122.956 | 20.057 |
| **Tumor stages** | | **Number** | **Mean** | | **95CI (low)** | **95CI (high)** | **SD** |
| Normal, T1 | | 40 | 119.75 | | 113.416 | 126.084 | 19.80514 |
| Normal, T2 | | 56 | 108.5714 | | 102.6947 | 114.4482 | 21.94444 |
| Normal, T3 | | 264 | 111.8561 | | 109.8207 | 113.8914 | 16.79563 |
| Normal, T4 | | 129 | 111.0853 | | 108.5342 | 113.6364 | 14.6437 |
| Luminal, T1 | | 53 | 123.9623 | | 117.8384 | 130.0861 | 22.21742 |
| Luminal, T2 | | 66 | 120.4545 | | 114.9757 | 125.9334 | 22.28707 |
| Luminal, T3 | | 302 | 121.8212 | | 119.7113 | 123.9311 | 18.63251 |
| Luminal, T4 | | 164 | 116.7073 | | 113.6706 | 119.744 | 19.69428 |
| Intratumoral, T1 | | 59 | 125.5932 | | 120.0911 | 131.0953 | 21.11309 |
| Intratumoral, T2 | | 69 | 121.4493 | | 116.2826 | 126.616 | 21.50755 |
| Intratumoral, T3 | | 322 | 122.4534 | | 120.3216 | 124.5852 | 19.44413 |
| Intratumoral, T4 | | 174 | 116.4368 | | 113.5055 | 119.368 | 19.58984 |
| Advancing Edge, T1 | | 60 | 126.1667 | | 120.9545 | 131.3788 | 20.17648 |
| Advancing Edge, T2 | | 63 | 124.7619 | | 119.9566 | 129.5672 | 19.08023 |
| Advancing Edge, T3 | | 324 | 121.9753 | | 119.9316 | 124.019 | 18.69884 |
| Advancing Edge, T4 | | 166 | 117.4096 | | 114.4731 | 120.3462 | 19.16217 |
| **One-way ANOVA, Luminal** | | | | | | | |
| t1 vs. t2: Diff= -4.144, 95%CI= -4.937 to 13.23, *p*= 0.642 | | | | | | | |
| t1 vs. t3: Diff= 3.14, 95%CI= -4.113 to 10.39, *p*= 0.680 | | | | | | | |
| **t1 vs. t4: Diff= 9.156, 95%CI= 1.441 to 16.87, *p*= 0.012** | | | | | | | |
| t2 vs. t3: Diff= -1.004, 95%CI= -7.798 to 5.790, *p*= 0.981 | | | | | | | |
| t2 vs. t4: Diff= 5.012, 95%CI= -2.274 to 12.30, *p*= 0.287 | | | | | | | |
| **t3 vs. t4: Diff= 6.017, 95%CI= 1.198 to 10.84, *p*= 0.007** | | | | | | | |
| **One-way ANOVA, Intratumoral** | | | | | | | |
| t1 vs. t2: Diff= 4.144, 95%CI= -4.937 to 13.23, *p*= 0.642 | | | | | | | |
| t1 vs. t3: Diff= 3.14, 95%CI= -4.113 to 10.39, *p*= 0.680 | | | | | | | |
| **t1 vs. t4: Diff= 9.156, 95%CI= 1.441 to 16.87, *p*= 0.012** | | | | | | | |
| t2 vs. t3: Diff= -1.004, 95%CI= -7.798 to 5.790, *p*= 0.981 | | | | | | | |
| t2 vs. t4: Diff= 5.012, 95%CI= -2.274 to 12.30, *p*= 0.287 | | | | | | | |
| **t3 vs. t4: Diff= 6.017, 95%CI= 1.198 to 10.84, *p*= 0.007** | | | | | | | |
| **One-way ANOVA, Advancing Edge** | | | | | | | |
| t1 vs. t2: Diff= 1.405, 95%CI= -7.430 to 10.24, *p*= 0.976 | | | | | | | |
| t1 vs. t3: Diff= 4.191, 95%CI= -2.692 to 11.07, *p*= 0.397 | | | | | | | |
| **t1 vs. t4: Diff= 8.757, 95%CI= 1.380 to 16.13, *p*= 0.012** | | | | | | | |
| t2 vs. t3: Diff= 2.787, 95%CI= -3.957 to 9.530, *p*= 0.711 | | | | | | | |
| **t2 vs. t4: Diff= 7.352, 95%CI= 0.1050 to 14.60, *p*= 0.045** | | | | | | | |
| t3 vs. t4: Diff= 4.566, 95%CI= -0.1090 to 9.240, *p*= 0.058 | | | | | | | |

**Supplementary Table S6. Survival statistical data.** For the survival analysis, cytoplasmic and nuclear tumor expression levels of each protein were normalised to the corresponding normal tissue control for each patient. Expression levels were categorized as either high or low in each cellular compartment based on the following criteria: low < mean expression, and high ≥ mean expression. The association between overall survival and protein expression (high vs. low) was assessed for each marker. Abbreviations: FLY: FLYWCH1,

GSK: GSK-3β, n: Nuclear, c: Cytoplasmic, max: Maximum, rat: Ratio, N: Number.

| **Variables** | **FLY. n. max** | **GSK. n. max** | **FLY. c. max** | **GSK. c. max** | **FLY. rat. n.** | **FLY. rat. c.** | **GSK. rat. N.** | **GSK. rat. C.** |
| --- | --- | --- | --- | --- | --- | --- | --- | --- |
| Mean | 100.112 | 36.304 | 146.429 | 125.419 | 0.696 | 1.216 | 2.756 | 1.125 |
| N | 667 | 579 | 686 | 585 | 426 | 442 | 246 | 357 |
| SD | 27.068 | 33.619 | 29.184 | 20.014 | 0.325 | 0.292 | 3.306 | 0.181 |
| 95%CI | 2.054 | 2.738 | 2.184 | 1.622 | 0.031 | 0.027 | 0.413 | 0.019 |
| High / low cutoff | n/a | n/a | n/a | n/a | 0.7 | 1.22 | 2.75 | 1.12 |
| High-Mean | n/a | n/a | n/a | n/a | 1.003 | 1.446 | 6.685 | 1.297 |
| Low-Mean | n/a | n/a | n/a | n/a | 0.536 | 1.025 | 1.130 | 0.988 |
| High-Count | n/a | n/a | n/a | n/a | 146 | 201 | 72 | 158 |
| Low-Count | n/a | n/a | n/a | n/a | 280 | 241 | 174 | 199 |
| High-Stdev | n/a | n/a | n/a | n/a | 0.378 | 0.259 | 3.808 | 0.121 |
| Low-Stdev | n/a | n/a | n/a | n/a | 0.259 | 0.477 | 0.528 | 0.421 |
| 95%CI-High | n/a | n/a | n/a | n/a | 0.061 | 0.036 | 0.879 | 0.019 |
| 95%CI-Low | n/a | n/a | n/a | n/a | 0.030 | 0.060 | 0.078 | 0.058 |

**Supplementary Table S7. *FLYWCH1* gRNAs and qRT-PCR primers sequences.**

| **Genes** | **Primer sequences 5'- 3'** |
| --- | --- |
| Human genes | |
| *FLYWCH1* | Forward: CTGGATGCAGCCCCTCAGT  Reverse: TTGGCGGCACTTCCAGTAC |
| *HPRT* | Forward: AGATGTGATGAAGGAGATG  Reverse: GTGTCAATTATATCTTCCA |
| *ACTB* | Forward: GCGCGGCTACAGCTTCA  Reverse: CTTAATGTCACGCACGATTTCC |
| *CD44* | Forward: AGAAGGTGTGGGCAGAAGAA  Reverse: AAATGCACCATTTCCTGAGA |
| *LGR5* | Forward: GACAACAGCAGTATGGACG  Reverse: GCATTACAAGTAAGTGCCAG |
| *CDX2* | Forward: AGCCAAGTGAAAACCAGGAC  Reverse: CCAGATTTTAACCTGCCTCTCA |
| *KRT20* | Forward: TGAAGAGCTGCGAAGTCAGAT  Reverse: TCCTCTCTCAGTCTCATACTTCAGTC |
| *CCND1* | Forward: GCTGGAGGTCTGCGAGGA  Reverse: CATCTTAGAGGCCACGAACA |
| *BMI1* | Forward: CCAGGGCTTTTCAAAAATGA  Reverse: CCGATCCAATCTGTTCTGGT |
| *CDH2* | Forward: GACAATGCCCCTCAAGTGTT  Reverse: CCATTAAGCCGAGTGATGGT |
| *FRZ8* | Forward: GGAGTGGGGTTACCTGTTGG  Reverse: GTAGCCGATGCCCTTACACA |
| *FRZ7* | Forward: TGTCGTTCTCTGTGCGAGC  Reverse: GAGCCGTCCGACGTGTTCT |
| *FRZ1* | Forward: GAAAGTGCAGTGTTCCGCTG  Reverse: CGAACTTGTTCATGAGCGCC |
| *WNT11* | Forward: TATCCGGCCTGTGAAGGACT  Reverse: GTCTTGTTGCACTGCCTGTC |
| *BCL9* | Forward: TGTCTTGATACCAGGAGGCCA  Reverse: TGGGCCCACATTCAGTCCTTTTT |
| *P21* | Forward: AGCTGAGGTGTGAGCAGC  Reverse: TTCTGACATGGCGCCTCC |
| *P53* | Forward: AAGTCTAGAGCCACCGTCCA  Reverse: GCAGTCTGGCCAATCCAGG |
| Mouse genes | |
| *math 1* | Forward: AGCTGTCCAAATATGAGACCCTACA  Reverse: GACATTGGGAGTCTGCAGCAA |
| *muc2* | Forward: TGGAGCCTGAAACACAATCACT  Reverse: ATGAAAATCAACAACCAGCTCATCT |
| *ctnnb1* | Forward: ATGGAGCCGGACAGAAAAGC  Reverse: TGGGAGGTGTCAACATCTTCTT |
| *cdx2* | Forward: CAAGGACGTGAGCATGTATCC  Forward: GTAACCACCGTAGTCCGGGTA |
| *ngn3* | Forward: GAAGCCGGCGCAAGAAG  Reverse: CATCCAGCGCCGAGTTG |
| *olfm4* | Forward: TGTGTCTCAGCCACTTTCCA  Reverse: TACTCGGACCGTCAGGTTCA |
| *lgr5* | Forward: CGGAGGAAGCGCTACAGAAT  Reverse: CTGGGTGGCACGTAGCTGAT |
| *cryp1* | Forward: CAGGTCCAGGCTGATCCTAT  Reverse: GCCTCCAAAGGAGACAGAAA |
| *pax4* | Forward: CTCGAATTGCCCAGCTAAAG  Reverse: TTACTGTGGGGACTGGGAAG |
| *atoh1* | Forward: GTAAGGAGAAGCGGCTGTG  Reverse: AGCCAAGCTCGTCCACTA |
| *tert* | Forward: CAGCCATACATGGGCCAGTTC  Reverse: ACAGGCTGCTGCTGCTCTCA |
| *lrig1* | Forward: TGTTTCCCGAACGGCCTGCGTA  Reverse: TGCTCAGACGGAGAGTCAGCAGTG |
| *chgA* | Forward: ACTTCCATGCAGGCTACAAAGC  Reverse: CTCTGTCTTTCCATCTCCATCCA |
| *gapdh* | Forward: TGAAGCAGGCATCTGAGGG  Reverse: CGAAGGTGGAAGAGTGGGAG |
| *FLYWCH1-*gRNA  (pLV-U6gRNA: hPGK-puro-2A-tBFP) | CCTGAAAGCCAGCAGATTTATGG (Sequence ID: NM_032296.3) |
| *FLYWCH1-*gRNA (pX549) | Forward: CACCGCAGGACGCAGTGCACTTCCT (BbsI site is underlined)  Reverse: AAACAGGAAGTGCACTGCGTCCTGC (BbsI site is underlined) |
